# Tirzepatide Associated With Improved Health‐Related Quality of Life in Adults With Obesity or Overweight in SURMOUNT‐4

**DOI:** 10.1002/oby.70011

**Published:** 2025-09-03

**Authors:** Theresa Hunter Gibble, Dachuang Cao, Madhumita Murphy, Irina Jouravskaya, Birong Liao, Harold Edward Bays

**Affiliations:** ^1^ Eli Lilly and Company Indianapolis Indiana USA; ^2^ Louisville Metabolic and Atherosclerosis Research Center Louisville Kentucky USA

**Keywords:** obesity, patient‐reported outcomes, tirzepatide health‐related quality of life

## Abstract

**Objective:**

In SURMOUNT‐4, participants with obesity or overweight regained substantial weight after tirzepatide withdrawal, whereas continued treatment resulted in additional weight reduction. We evaluated the effect of continued tirzepatide treatment on health‐related quality of life (HRQoL) in SURMOUNT‐4.

**Methods:**

Participants who achieved tirzepatide maximum tolerated dose (MTD; 10 or 15 mg; *N* = 670) during a 36‐week (W) lead‐in period were randomized (1:1) to continue receiving tirzepatide MTD or switch to placebo through W88. HRQoL was assessed using patient‐reported outcomes (PROs: SF‐36v2, IWQOL‐Lite‐CT, EQ‐5D‐5L). Post hoc analysis included changes in PROs by weight reduction categories and baseline Patient Global Impression of Status for physical activity response categories among tirzepatide‐treated participants and by weight regain categories in the placebo group.

**Results:**

Tirzepatide treatment was associated with improved PROs from W0 to W36, and these improvements were maintained from W36 to W88 with continued tirzepatide treatment versus placebo. Tirzepatide‐treated participants with greater weight reduction and those with physical function limitations at baseline showed numerically greater improvements in PROs. Greater weight regain among participants who switched to placebo was associated with worsening of PROs.

**Conclusions:**

In participants with overweight or obesity, continued tirzepatide treatment was associated with maintaining HRQoL improvement, while treatment withdrawal resulted in worsening of HRQoL.

**Trial Registration:** ClinicalTrials.gov identifier NCT04660643.

**Study Importance**

- What is already known?
  ○ Following an initial open‐label tirzepatide lead‐in treatment period in participants with obesity or overweight, the phase 3 SURMOUNT‐4 (NCT04660643) trial evaluated the effect of continued treatment with tirzepatide maximum tolerated dose (MTD; 10 or 15 mg) versus placebo on the maintenance of weight reduction.
  ○ Withdrawing tirzepatide resulted in substantial regain of prior weight reduction; continued treatment with tirzepatide led to sustained and additional weight reduction.
- What does this study add?
  ○ We evaluated the association between continued treatment with tirzepatide MTD and patient‐reported health‐related quality of life (HRQoL) among SURMOUNT‐4 participants.
  ○ Improvements in general and weight‐related quality of life achieved during the tirzepatide lead‐in period were maintained in participants who continued to receive tirzepatide MTD and worsened among participants who switched to placebo, resulting in statistically significant differences between the two groups. Among tirzepatide‐treated participants, limitations in physical function at baseline and greater weight reduction were associated with numerically greater improvements in HRQoL. Among participants who switched to placebo, weight regain was associated with worsening of HRQoL.
- How might these results change the direction of research or the focus of clinical practice?
  ○ The study findings highlight the importance of continuing pharmacotherapy to preserve HRQoL benefits in participants with obesity.

## 1 Introduction

Obesity is a chronic, complex, highly prevalent, and relapsing disease associated with numerous complications such as type 2 diabetes (T2D), cardiovascular diseases, obstructive sleep apnea, osteoarthritis, and mental health disorders [1]. According to the National Health and Wellness Survey, high body mass index (BMI) is associated with lower health‐related quality of life (HRQoL) and reduced work productivity [2]. HRQoL encompasses physical and psychosocial health domains, which are important measures for understanding an individual's overall health perception. For people living with obesity, HRQoL, particularly in terms of physical functioning, is a significant concern. Therefore, it is recommended that HRQoL be assessed using patient‐reported outcomes (PROs) as a key measure in clinical trials [3].

American Diabetes Association's Standard of Care treatment guidelines recommend 3%–7% weight reduction for improvement in obesity‐related complications, which is typically associated with enhanced daily functioning and psychosocial well‐being in people with obesity [4, 5]. Long‐term adherence to obesity management programs is often challenging, and weight regain is common [6, 7, 8]. Treatment with obesity pharmacotherapy, as an adjunct to lifestyle interventions, is recommended for sustained weight reduction and improving HRQoL [5]. The impact of pharmacotherapy on HRQoL can be examined using generic or disease‐specific measures, focusing on obesity [9].

Tirzepatide is a once‐weekly glucose‐dependent insulinotropic polypeptide (GIP) and glucagon‐like peptide‐1 (GLP‐1) receptor agonist that selectively binds to and activates both the GIP and GLP‐1 receptors [10]. Tirzepatide is approved in many countries, including the United States (US), as an adjunct to diet and physical activity for the treatment of adults with T2D, chronic weight management, and obstructive sleep apnea [10, 11].

In the phase 3 SURMOUNT‐4 (NCT04660643) trial, participants with obesity or overweight who completed the 36‐week tirzepatide lead‐in period continued treatment with tirzepatide maximum tolerated dose (MTD; 10 or 15 mg) over the next 52 weeks, resulting in an additional mean weight reduction of 5.5%, whereas participants who switched to placebo experienced a mean weight regain of 14.0% (treatment regimen estimand). The mean weight reduction during the entire study (Weeks 0–88) was 25.3% with tirzepatide MTD and 9.9% with placebo [12]. Here, we evaluate the association between continued treatment with tirzepatide MTD and patient‐reported HRQoL among SURMOUNT‐4 participants.

## 2 Methods

### 2.1 Study Design and Population

SURMOUNT‐4 was a phase 3, multicenter (conducted at 70 sites in four countries), randomized withdrawal study with a 36‐week open‐label tirzepatide lead‐in period followed by a 52‐week, double‐blind, placebo‐controlled period [12]. Eligible participants were adults (≥ 18 years old) who had obesity (BMI ≥ 30 kg/m^2^) or overweight (BMI ≥ 25–< 30 kg/m^2^) with at least one obesity‐related complication (i.e., hypertension, dyslipidemia, obstructive sleep apnea, or cardiovascular disease) and a history of at least one self‐reported unsuccessful dietary effort to lose body weight. People with type 1 diabetes, T2D, prior surgical treatment for obesity, or treatment with an obesity medication within 3 months prior to enrollment were excluded. The detailed study design and endpoints have been published previously [12]. The study protocol was approved by the ethical review board and adhered to the principles outlined in the Declaration of Helsinki, the Council of International Organizations of Medical Sciences International Ethical Guidelines, and Good Clinical Practice guidelines. All participants provided written informed consent before study enrollment.

### 2.2 Randomization and Treatments

In SURMOUNT‐4, tirzepatide was subcutaneously administered once weekly with a starting dose of 2.5 mg. Tirzepatide dose escalation was implemented during the 36‐week lead‐in period, with doses increased by 2.5 mg every four weeks until a MTD of 10 mg or 15 mg was achieved. Participants who attained tirzepatide MTD were randomly assigned in a 1:1 ratio to either continue receiving tirzepatide MTD or switch to placebo for an additional 52 weeks (double‐blind treatment period). Throughout the study, all participants received study treatment as an adjunct to lifestyle counseling (500 kcal/day deficit diet and at least 150 min of physical activity/week).

### 2.3 Study Outcomes and Assessments

The effect of tirzepatide on HRQoL was assessed using the following PROs during the tirzepatide lead‐in period (Weeks 0–36), the double‐blind treatment period (Weeks 36–88), and the entire study period (Weeks 0–88). With the randomized withdrawal design of SURMOUNT‐4, the primary focus was to demonstrate that tirzepatide MTD was superior to placebo for change in HRQoL from randomization (Week 36) at 88 weeks. A list of prespecified and post hoc endpoints and assessments is provided in Table S1.

#### 2.3.1 Short Form‐36 Version 2 Health Survey (SF‐36v2) Acute Form, 1‐Week Recall Version [13]

The SF‐36v2 is a 36‐item validated PRO measure used to assess generic HRQoL and health status. It consists of eight domains: Physical Functioning; Role‐Physical; Bodily Pain; General Health; Vitality; Social Functioning; Role‐Emotional; and Mental Health. Each domain is scored individually, and these scores are combined into two component summary scores: Physical Component Summary (PCS) and Mental Component Summary (MCS). The Physical Functioning domain assesses limitations due to health “now,” while the remaining domains assess functioning “in the last week.” Items are answered on Likert scales of varying lengths (3‐, 5‐, or 6‐point). A 3‐ to 5‐point increase in PCS or MCS score is considered clinically meaningful [14]. The domain and component summary scores are norm‐based to the 2009 US general population, with a mean of 50 and a standard deviation (SD) of 10. Higher scores indicate better HRQoL.

#### 2.3.2 Impact of Weight on Quality of Life‐Lite‐Clinical Trials Version (IWQOL‐Lite‐CT) [15]

Weight‐related quality of life was assessed using IWQOL‐Lite‐CT, a 20‐item validated obesity‐specific PRO instrument developed in accordance with US Food and Drug Administration (FDA) guidance for use in clinical trials. This instrument assesses two primary domains of obesity‐related and health‐related quality of life: Physical composite (7 items) and Psychosocial composite (13 items). A 5‐item subset of the Physical composite, the Physical Function composite, is also assessed. Items in the Physical Function composite describe physical impacts related to general and specific physical activities. All items are rated on either a 5‐point frequency (“never” to “always”) or a 5‐point truth (“not at all true” to “completely true”) scale. The overall score range is from 0 to 100 with higher scores indicating better functioning. A change of 13.5–16.6 points in IWQOL‐Lite‐CT scores is considered clinically meaningful [16].

#### 2.3.3 EQ‐5D‐5L [17]

The EQ‐5D‐5L is a standardized five‐dimension instrument that yields a simple descriptive profile and a single index value for health status. It comprises five dimensions of health: mobility; self‐care; usual activities; pain/discomfort; and anxiety/depression. Each dimension was scored on five levels (no problems, slight problems, moderate problems, severe problems, or unable to perform/extreme problems). The Health State Index value is derived using a formula that assigns weights to the levels in each dimension. This index value ranges from < 0 to 1, where 0 is a health state equivalent to death; negative values are valued as worse than dead, and 1 is considered perfect health [18]. Additionally, the EQ Visual Analogue Scale (VAS) records the participant's self‐rated health status on a vertical graduated scale of 0–100. A 0.03‐point difference in EQ‐5D index score and 10‐point difference on the EQ‐VAS are considered minimal clinically important difference [19, 20, 21].

#### 2.3.4 Patient Global Impression of Status (PGIS) for Physical Activity

The PGIS for physical activity was specifically developed for the SURMOUNT‐4 study. This is a participant‐rated assessment of current limitations on physical activity due to health and is rated on a 5‐point scale ranging from “not at all limited” to “extremely limited.” Participants with responses of “moderately,” “very much,” or “extremely” limited were classified as having physical function limitations at baseline.

Study endpoints (prespecified from Weeks 36 to 88 and Weeks 0 to 88 and post hoc during Weeks 0 to 36) included changes in [1] SF‐36v2 norm‐based scores, PCS, MCS, and domain (Physical Functioning, Role‐Physical, Bodily Pain, General Health, Vitality, Social Functioning, Role‐Emotional, and Mental Health) scores; [2] IWQOL‐Lite‐CT Total and composite scores (Physical Function, Physical, and Psychosocial); and [3] EQ‐5D‐5L Health State Index (UK) and EQ‐VAS scores. Among participants who had physical function limitations at baseline, changes in SF‐36v2 Physical Functioning domain score and IWQOL‐Lite‐CT Physical Function composite score were assessed. The number and proportion of participants endorsing each PGIS for physical activity response category on a 5‐point scale (“not at all limited” to “extremely limited”) were also reported.

Additionally, post hoc analyses included the proportion of participants achieving meaningful within‐participant change in SF‐36v2 Physical Functioning domain score (improvement of ≥ 5.76). The association of percentage weight reduction categories (≥ 5%, ≥ 10%, ≥ 15%, ≥ 20%, ≥ 25%, and ≥ 30%) and baseline PGIS status (presence or absence of physical function limitations) with PROs (SF‐36v2, IWQOL‐Lite‐CT, and EQ‐5D‐5L) was assessed among tirzepatide‐treated participants. The association between percentage weight regain categories (< 25%, 25%–< 50%, 50%–< 75%, and ≥ 75%) and PROs (SF‐36v2, IWQOL‐Lite‐CT, and EQ‐5D‐5L) was assessed during Weeks 36 to 88 among participants in the placebo group who achieved ≥ 10% weight loss at Week 36.

### 2.4 Statistical Analyses

Statistical analyses were conducted using the SAS version 9.4 software. PROs were analyzed in the efficacy analysis set (all randomly assigned participants who were exposed to at least one dose of the study drug, excluding data after study drug discontinuation).

All participants received tirzepatide during the 36‐week lead‐in period per study design. The results for this period are presented for tirzepatide MTD and placebo groups based on the treatment assignment of participants at randomization (Week 36), for the subsequent 52‐week double‐blind treatment period.

For continuous outcomes, the least squares mean (LSM) difference for pairwise comparison (tirzepatide MTD vs. placebo) was calculated using an analysis of covariance (ANCOVA) model with the last observation carried forward for missing data imputation. The ANCOVA model was used with treatment, stratification factors (country, sex, weight loss at Week 36, and tirzepatide MTD at Week 36), and PRO scores at baseline (Week 0) or randomization (Week 36) as covariates. No multiplicity adjustments were made for the reported PRO measures. The *p* values reported are unadjusted for multiple testing and should not be interpreted as confirmatory. The counts and percentages of participants endorsing each PGIS response category were summarized by nominal visit and by treatment.

For post hoc analyses, the mean changes in PROs by percentage weight reduction categories and by baseline PGIS status (presence or absence of physical function limitations) were summarized descriptively among tirzepatide‐treated participants. The mean changes in PROs by percentage weight regain categories were summarized descriptively in participants receiving placebo.

## 3 Results

### 3.1 Patient Disposition and Baseline Characteristics

A total of 670 participants who attained tirzepatide MTD (15 mg: *n* = 621 [92.7%]; 10 mg: *n* = 49 [7.3%]) were randomly assigned at Week 36 to either continue receiving tirzepatide MTD (*n* = 335) or switch to placebo (*n* = 335) (Table 1). At baseline (Week 0), participants had a mean body weight of 107.3 kg, BMI of 38.4 kg/m^2^, and obesity duration of 15.5 years.

**TABLE 1:** Baseline demographics and clinical characteristics.

Characteristics	Week 0 (start of tirzepatide lead‐in treatment period), *N* = 670	Week 36 (randomization)	
		Tirzepatide MTD, *N* = 335	Placebo, *N* = 335
Age (years)	47.7 (12.6)	49.2 (12.8)	48.1 (12.4)
Female, *n* (%)	473 (70.6)	236 (70.4)	237 (70.7)
Duration of obesity^a^ (years)	15.5 (11.8)	15.9 (12.1)	15.2 (11.4)
Body weight (kg)	107.3 (22.3)	84.6 (19.8)	85.8 (22.3)
BMI (kg/m^2^)	38.4 (6.6)	30.3 (6.0)	30.7 (6.8)
BMI category (kg/m^2^), *n* (%)			
< 25	—	59 (17.6)	63 (18.8)
≥ 25 to < 30	18 (2.7)	122 (36.4)	120 (35.8)
≥ 30 to < 35	212 (31.6)	88 (26.3)	75 (22.4)
≥ 35 to < 40	215 (32.1)	41 (12.2)	43 (12.8)
≥ 40	225 (33.6)	25 (7.5)	34 (10.1)
Waist circumference (cm)	115.2 (14.5)	96.8 (14.1)	98.2 (16.0)
Short Form‐36 Version 2 Health Survey Acute Form (SF‐36v2) scores^b^ (general quality of life survey)			
Physical Component Summary (PCS) score	48.9 (7.6)	54.7 (5.5)	54.3 (6.8)
Mental Component Summary (MCS) score	52.5 (8.0)	54.5 (6.5)	54.5 (6.9)
Physical Functioning domain	47.6 (8.2)	53.4 (5.9)	53.4 (6.4)
Role‐Physical domain	50.1 (7.8)	54.6 (4.9)	53.7 (6.8)
Bodily Pain domain	50.4 (9.0)	54.3 (6.9)	54.4 (8.2)
General Health domain	50.7 (8.0)	56.6 (6.3)	56.2 (7.2)
Vitality domain	52.3 (8.4)	57.2 (7.3)	57.1 (7.7)
Social Functioning domain	51.7 (7.5)	54.5 (5.5)	53.9 (6.4)
Role‐Emotional domain	49.5 (8.9)	52.5 (6.9)	52.2 (7.5)
Mental Health domain	52.6 (7.8)	54.8 (6.5)	55.3 (6.7)
Impact of Weight on Quality of Life‐Lite‐Clinical Trials Version^c^ (IWQOL‐Lite‐CT; weight‐related quality of life questionnaire)			
Total score	58.0 (22.4)	81.5 (15.2)	82.0 (16.2)
Physical Function composite score	59.1 (24.5)	81.0 (17.0)	81.7 (18.2)
Physical composite score	58.8 (23.6)	79.8 (16.6)	80.0 (17.8)
Psychosocial composite score	57.5 (24.3)	82.4 (16.3)	83.0 (16.8)
EQ‐5D‐5L (health status questionnaire)			
Health State Index (UK)	0.8 (0.2)	0.9 (0.1)	0.9 (0.1)
EQ Visual Analogue Scale	73.9 (17.0)	85.7 (10.5)	84.8 (12.5)

*Note*: Data are presented as mean (SD), unless specified otherwise. The baseline scores for SF‐36v2 and IWQOL‐Lite‐CT are reported for the efficacy analysis set at Week 0 (lead‐in) and Week 36 (randomization).

Abbreviations: MTD, maximum tolerated dose; *n*, number of participants in the specified category; *N*, total number of participants.

^a^ Duration of obesity was self‐reported by participants.

^b^ The SF‐36v2 scores are norm‐based, that is, scores transformed to a scale in which the 2009 US general population has a mean score of 50 and an SD of 10. Higher scores indicate better health.

^c^ Scores are transformed to a scale of 0 to 100, with higher scores reflecting better levels of functioning.

At randomization (Week 36), demographics and clinical characteristics were similar between the tirzepatide MTD and placebo groups, with a mean body weight of 85.2 kg and a mean BMI of 30.5 kg/m^2^ (Table 1).

### 3.2 Patient‐Reported Outcomes

#### 3.2.1 Short Form‐36 Version 2 Health Survey Acute Form

**FIGURE 1:**
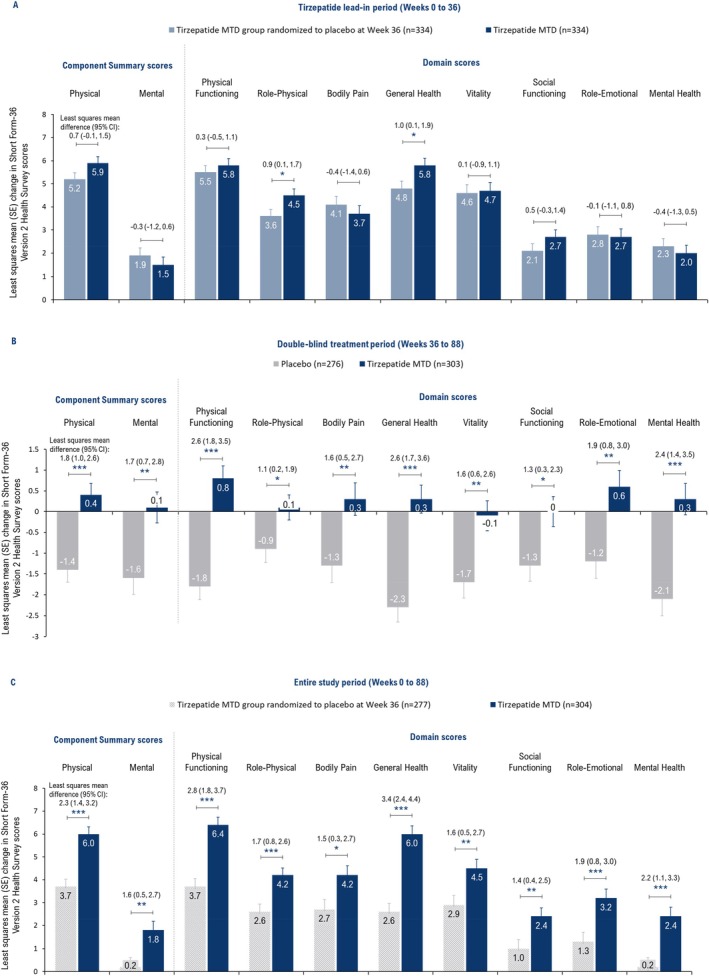
Effect of tirzepatide versus placebo on general quality of life measured by Short Form‐36 Version 2 Health Survey Acute Form (SF‐36v2). Data are presented as least squares mean change from baseline (Week 0) or randomization (Week 36) using ANCOVA with last observation carried forward for the efficacy analysis set. The SF‐36v2 scores are norm‐based, that is, scores transformed to a scale in which the 2009 US general population has a mean score of 50 and standard deviation of 10. A higher score indicates better health. *p < 0.05, **p < 0.01, ***p < 0.001 versus placebo. MTD, maximum tolerated dose; *n*, number of participants with baseline and post‐baseline value at the specified time point; SE, standard error. [Color figure can be viewed at wileyonlinelibrary.com]

Improvements in SF‐36v2 PCS, MCS, and domain scores observed during the tirzepatide lead‐in period (Weeks 0–36; Figure 1A) were maintained from Weeks 36 to 88 in participants who continued treatment with tirzepatide MTD, but worsened with placebo, resulting in statistically significant differences between the groups (Figure 1B; PCS: *p* < 0.001; MCS: *p* < 0.01; and domain scores: *p* < 0.001 for Physical Functioning, General Health, and Mental Health, *p* < 0.01 for Bodily Pain, Vitality, and Role‐Emotional, and *p* < 0.05 for Role‐Physical and Social Functioning). During the study period (Weeks 0–88), tirzepatide was associated with significant improvements versus placebo in SF‐36v2 PCS (*p* < 0.001), MCS (*p* < 0.01), and all the domain scores (*p* < 0.05) (Figure 1C).

**FIGURE 2:**
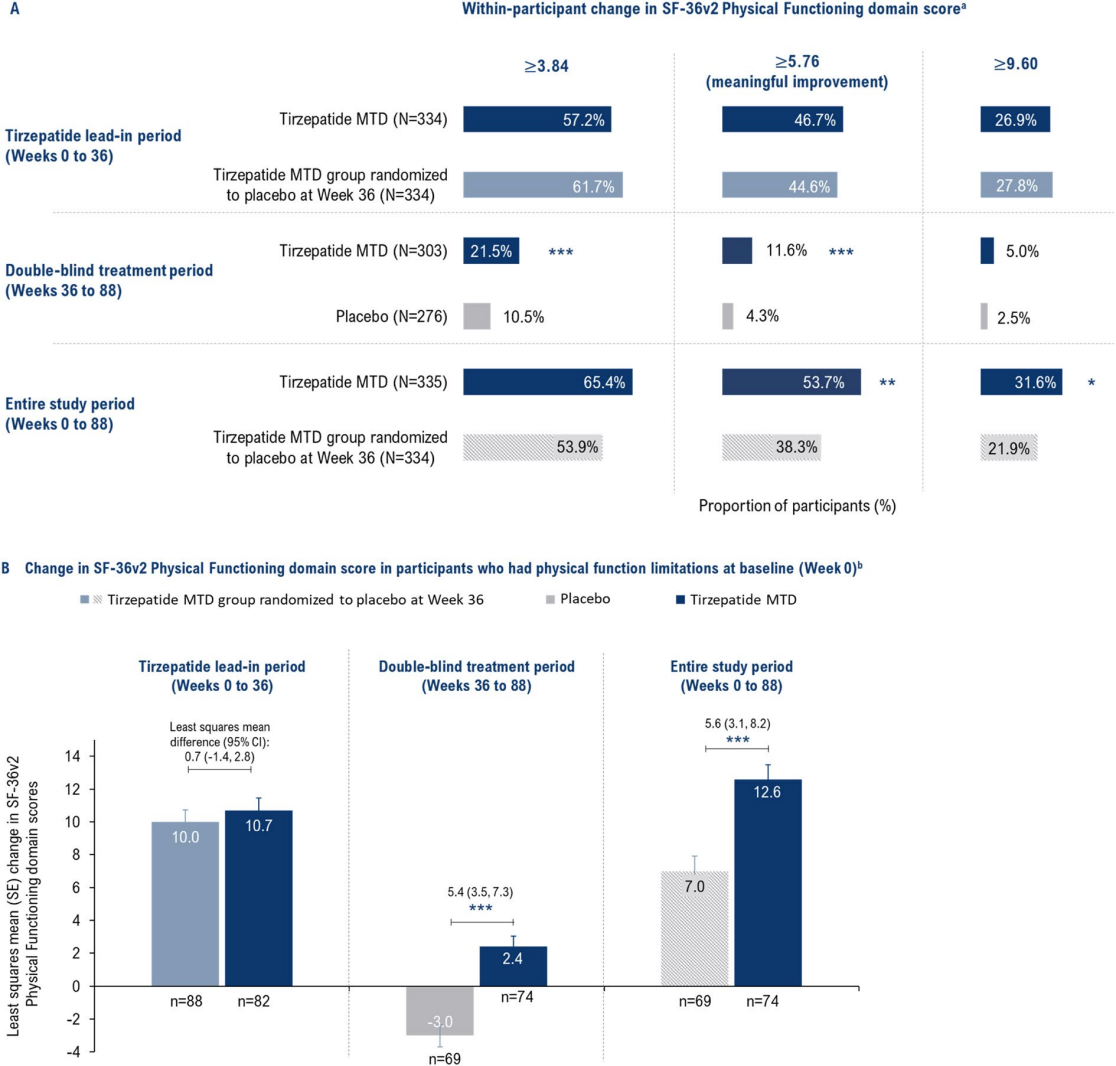
Effect of tirzepatide versus placebo on the Physical Functioning domain score of the Short Form‐36 Version 2 Health Survey Acute Form (SF‐36v2; general quality of life survey). Data are presented for the efficacy analysis set: (A) proportion of participants achieving within‐treatment change from baseline (Week 0) or randomization (Week 36); (B) least square mean change from baseline (Week 0) or randomization (Week 36) using ANCOVA with the last observation carried forward. The SF‐36v2 scores are norm‐based, that is, scores transformed to a scale in which the 2009 US general population has a mean score of 50 and standard deviation of 10. A higher score indicates better health. *p < 0.05, **p < 0.01, ***p < 0.001 versus placebo. ^a^A meaningful within‐participant change from the start of open‐label lead‐in (Week 0) or randomization (Week 36) in SF‐36v2 Physical Functioning domain score was defined as an increase of ≥ 5.76 (range 3.84–9.60) using anchor‐based and distribution‐based methods [22]. ^b^Participants with physical function limitations at baseline were those who rated their physical function as “moderately limited,” or “very much limited,” or “extremely limited” in the Patient Global Impression of Status for physical activity. MTD, maximum tolerated dose; *N*, number of participants with baseline and post‐baseline value at the specified time point; *n*, number of participants with physical function limitations at baseline who had baseline and post‐baseline value at the specified time point; SE, standard error. [Color figure can be viewed at wileyonlinelibrary.com]

At the end of the 36‐week lead‐in treatment with tirzepatide, the proportion of participants achieving meaningful within‐participant change in the SF‐36v2 Physical Functioning domain scores (improvement of ≥ 5.76; norm‐based) was similar between those subsequently randomized to tirzepatide MTD (46.7%) and placebo (44.6%) at Week 36. More participants achieved meaningful within‐participant change in the SF‐36v2 Physical Functioning domain score with tirzepatide MTD than with placebo during Weeks 36 to 88 (11.6% vs. 4.3%) and Weeks 0 to 88 (53.7% vs. 38.3%) (Figure 2A).

Among participants who had physical function limitations at baseline (Week 0), the improvements in SF‐36v2 Physical Functioning domain scores during the tirzepatide lead‐in period (Weeks 0–36) were comparable between those randomized to continue tirzepatide MTD (LSM: 10.7; *n* = 82) and those randomized to placebo (10.0; *n* = 88) groups at Week 36 (Figure 2B). Continued treatment with tirzepatide MTD (*n* = 74) versus placebo (*n* = 69) resulted in sustained improvements in SF‐36v2 Physical Functioning domain scores from Weeks 36 to 88 (LSM difference vs. placebo: 5.4; *p* < 0.001) and Weeks 0–88 (5.6; *p* < 0.001) among these participants (Figure 2B).

#### 3.2.2 Impact of Weight on Quality of Life‐Lite‐Clinical Trials Version

**FIGURE 3:**
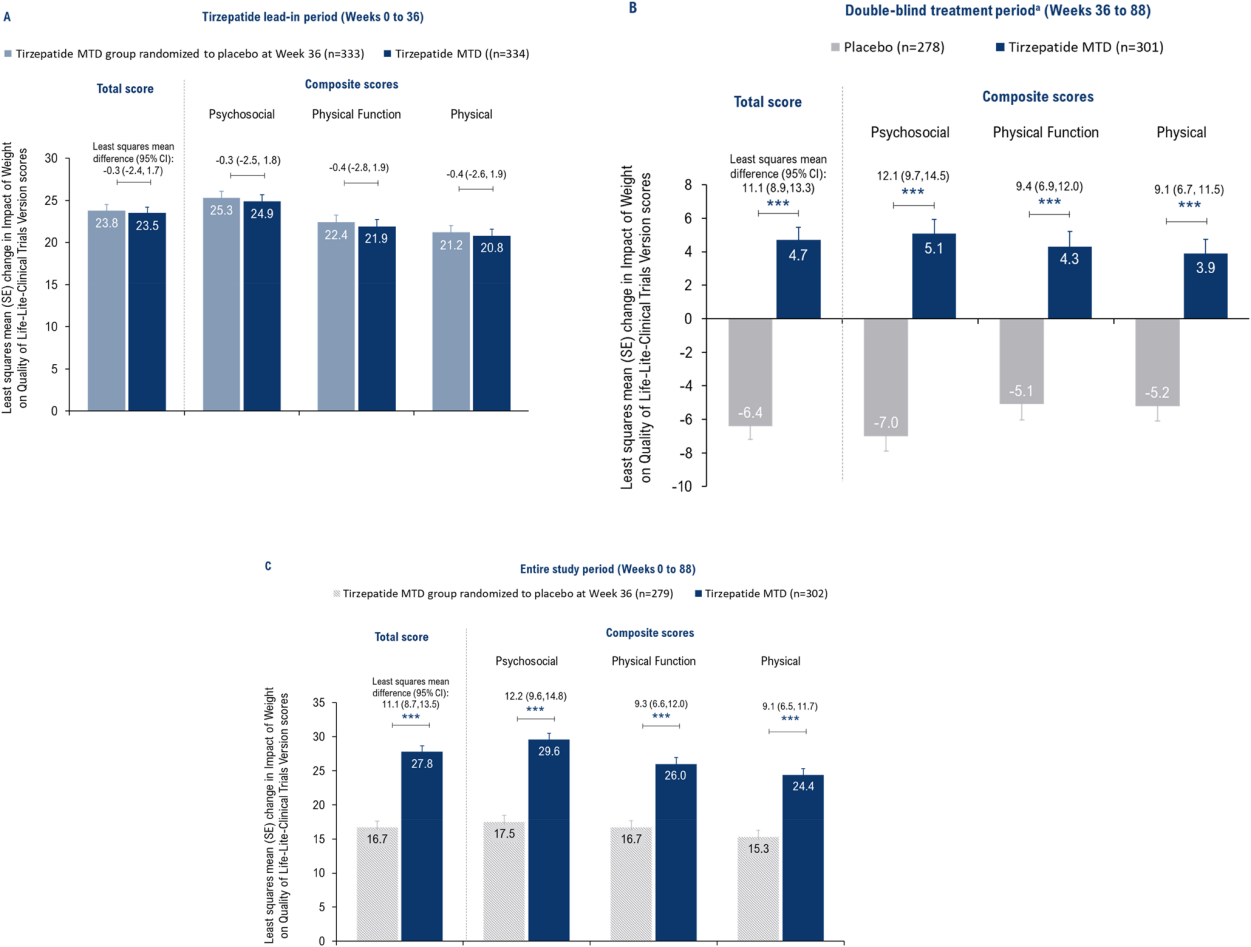
Effect of tirzepatide versus placebo on weight‐related quality of life measured by Impact of Weight on Quality of Life‐Lite‐Clinical Trials Version (IWQOL‐Lite‐CT). Data are presented as least squares mean change from baseline (Week 0) or randomization (Week 36) using ANCOVA with the last observation carried forward for the efficacy analysis set. ***p < 0.001 versus placebo. ^a^For tirzepatide MTD and placebo groups, within‐treatment change from Weeks 36 to 88 was significant for total and composite (Psychosocial, Physical Function, and Physical) scores (all *p* < 0.001). MTD, maximum tolerated dose; *n*, number of participants with baseline and post‐baseline value at the specified time point; SE, standard error. [Color figure can be viewed at wileyonlinelibrary.com]

During the tirzepatide lead‐in period (Weeks 0–36), the tirzepatide MTD and placebo groups showed similar improvements in IWQOL‐Lite‐CT Total (LSM: 23.5 and 23.8) and composite (Psychosocial: 24.9 and 25.3; Physical Function: 21.9 and 22.4; and Physical: 20.8 and 21.2) scores (Figure 3A). During Weeks 36 to 88, continued treatment with tirzepatide showed additional improvements, while switching to placebo showed worsening, with significant differences between groups (IWQOL‐Lite‐CT Total score LSM difference vs. placebo: 11.1, Psychosocial composite score: 12.1, Physical Function composite score: 9.4, and Physical composite score: 9.1; all *p* < 0.001; Figure 3B). Throughout the study period, tirzepatide treatment was associated with improvements in IWQOL‐Lite‐CT Total score and all the composite scores compared to placebo (all *p* < 0.001; Figure 3C).

**FIGURE 4:**
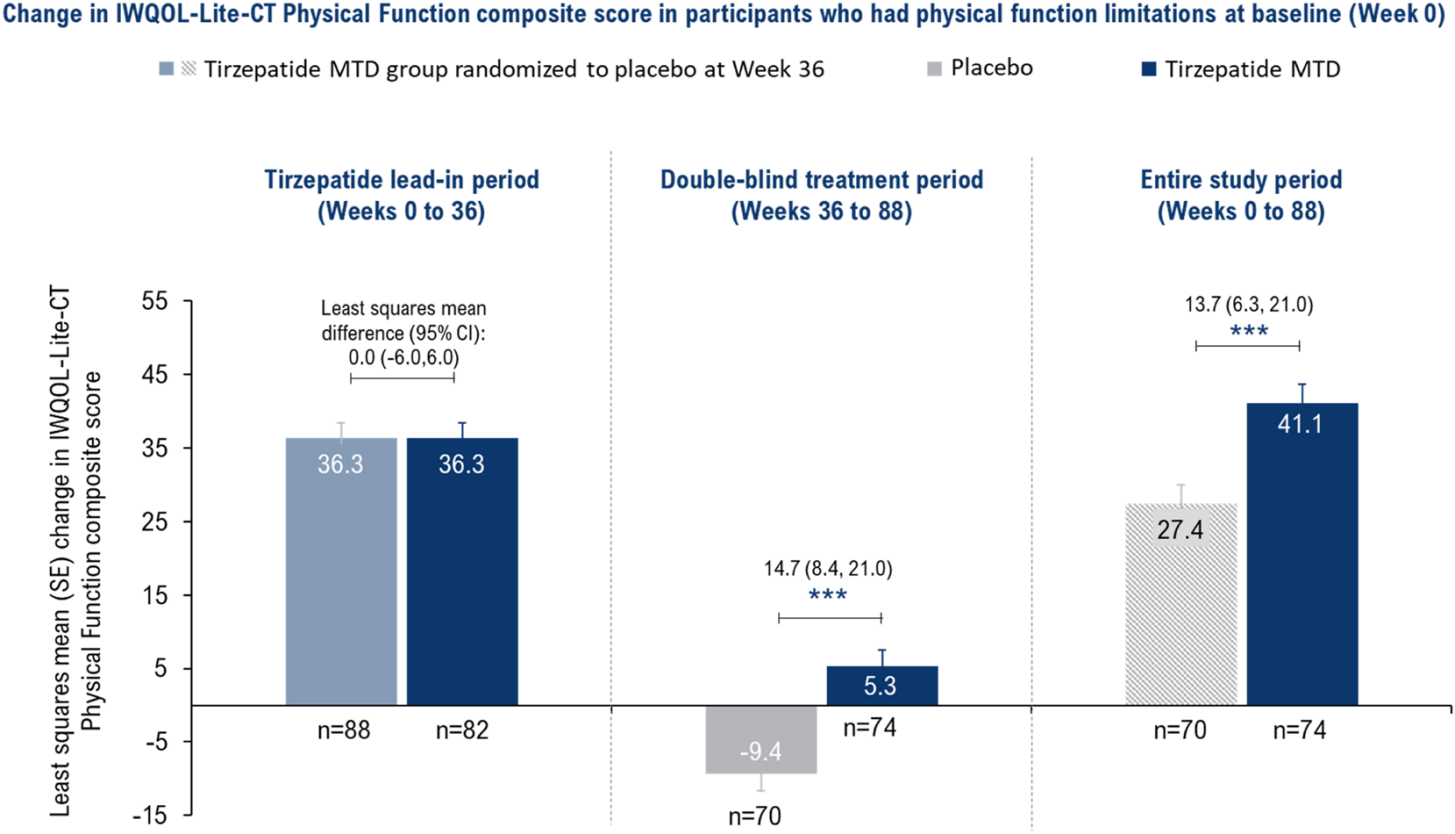
Effect of tirzepatide versus placebo on the Physical Function composite score of the Impact of Weight on Quality of Life‐Lite‐Clinical Trials Version (IWQOL‐Lite‐CT; weight‐related quality of life questionnaire). Data are presented as least squares mean change from baseline (Week 0) or randomization (Week 36) using ANCOVA with the last observation carried forward for the efficacy analysis set. ***p < 0.001 versus placebo. Participants with physical function limitations at baseline were those who rated their physical function as “moderately limited,” or “very much limited,” or “extremely limited” in the Patient Global Impression of Status for physical activity. MTD, maximum tolerated dose; *n*, number of participants with physical function limitations at baseline who had baseline and post‐baseline value at the specified time point; SE, standard error. [Color figure can be viewed at wileyonlinelibrary.com]

Among participants with physical function limitations at baseline, the improvement in IWQOL‐Lite‐CT Physical Function composite score during the tirzepatide lead‐in period (Weeks 0–36) was the same for the tirzepatide MTD (*n* = 82) and placebo (*n* = 88) groups (LSM: 36.3 for both; Figure 4). Continued treatment with tirzepatide MTD (*n* = 74) versus placebo (*n* = 70) led to sustained improvements in IWQOL‐Lite‐CT Physical Function composite scores from Weeks 36 to 88 (LSM difference vs. placebo: 14.7; *p* < 0.001), and Weeks 0 to 88 (13.7; *p* < 0.001) in these participants (Figure 4).

#### 3.2.3 EQ‐5D‐5L Scores

**FIGURE 5:**
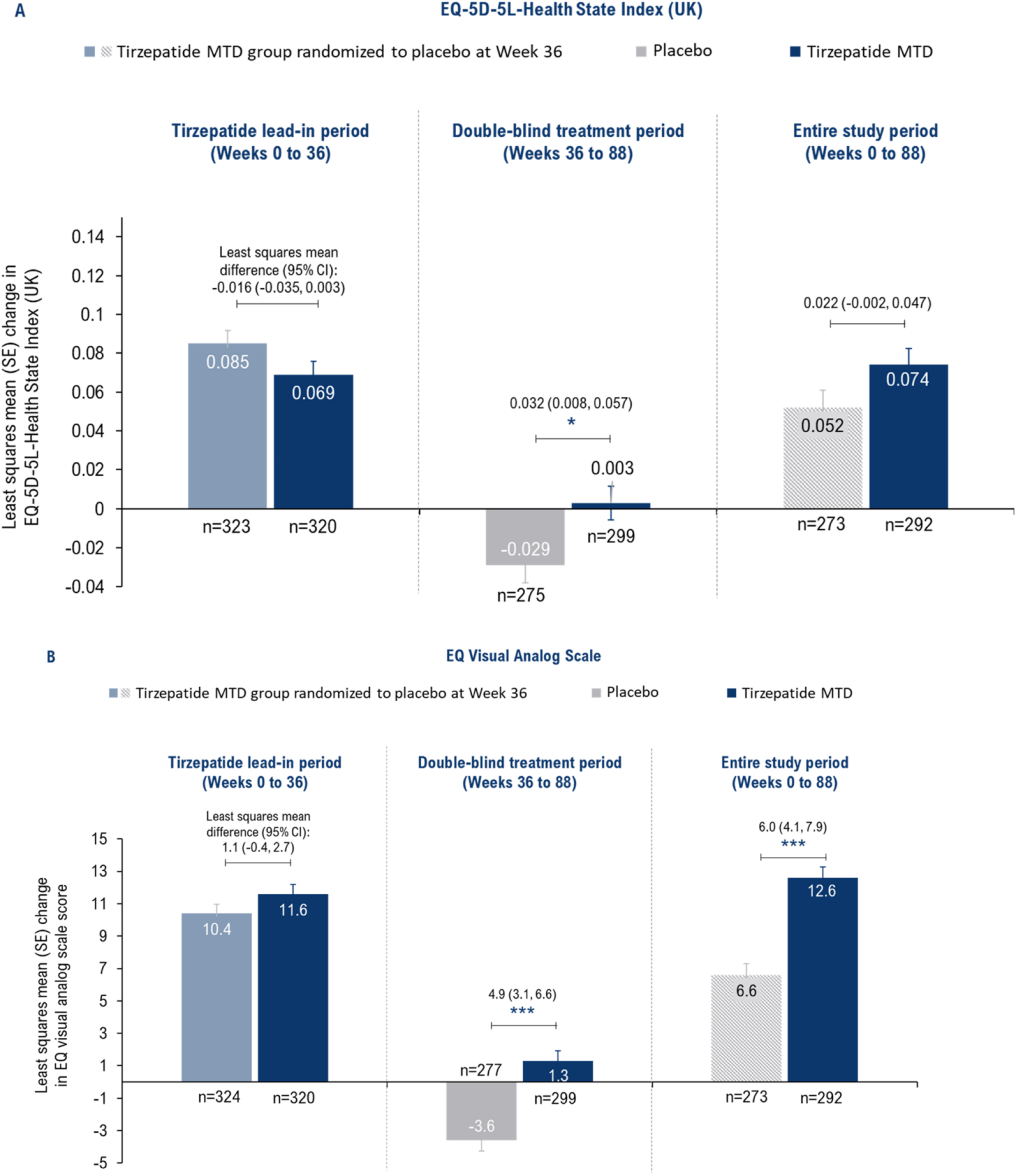
Effect of tirzepatide versus placebo on health status measured by EQ‐5D‐5L. Data are presented as least squares mean change from baseline (Week 0) or randomization (Week 36) using ANCOVA with the last observation carried forward for the efficacy analysis set. *p < 0.05, ***p < 0.001 versus placebo. Diff, difference; MTD, maximum tolerated dose; *n*, number of participants with baseline and post‐baseline value at the specified time point; SE, standard error. [Color figure can be viewed at wileyonlinelibrary.com]

The improvement from baseline in EQ‐5D‐5L‐Health State Index (UK) scores during tirzepatide lead‐in period (Weeks 0–36) and study period (Weeks 0–88) was comparable between the treatment groups (Figure 5A). From Weeks 36 to 88, improvement in EQ‐5D‐5L‐Health State Index (UK) scores was maintained in the tirzepatide group, whereas worsening was seen in the placebo group (*p* < 0.05; Figure 5A).

The improvement in EQ VAS scores was similar between the tirzepatide MTD (LSM: 11.6) and placebo (10.4) groups during the tirzepatide lead‐in period (Weeks 0–36), and these improvements were maintained with tirzepatide treatment from Weeks 36 to 88 (LSM difference vs. placebo: 4.9; *p* < 0.001) and Weeks 0 to 88 (6.0; *p* < 0.001) (Figure 5B).

#### 3.2.4 Patient Global Impression of Status for Physical Activity

Among the participants who had “moderately limited” to “extremely limited” physical activity at baseline (placebo: *n* = 88; tirzepatide MTD: *n* = 82), most reported an improvement at Week 36 in both treatment groups (placebo: *n* = 81, 92.0%; tirzepatide MTD: *n* = 78, 95.1%; Table 2). At Week 36 (randomization), a majority of the participants in both treatment groups (75.5%; *n* = 253 for both) reported that their physical activity was “not at all limited.” However, decline in physical activity at Week 88 was higher in participants who switched to placebo than in those who continued to receive tirzepatide MTD (Table 2).

**TABLE 2:** Shift table of Patient Global Impression of Status for physical activity response categories. [Color table can be viewed at wileyonlinelibrary.com]

Tirzepatide lead‐in period (Weeks 0–36)—Randomized population								
		Response group at Week 36, *n* (%)						
Treatment	Response group at Week 0, *n* (%)	Not at all limited	A little limited	Moderately limited	Very much limited	Extremely limited	Missing	Total
Tirzepatide MTD group randomized to placebo at Week 36 (*N* = 335)	Not at all limited	124 (37.0)	13 (3.9)	1 (0.3)	0	2 (0.6)	0	140 (41.8)
	A little limited	76 (22.7)	26 (7.8)	4 (1.2)	1 (0.3)	0	0	107 (31.9)
	Moderately limited	46 (13.7)	17 (5.1)	4 (1.2)	1 (0.3)	0	0	68 (20.3)
	Very much limited	9 (2.7)	4 (1.2)	3 (0.9)	1 (0.3)	1 (0.3)	0	18 (5.4)
	Extremely limited	0	1 (0.3)	0	1 (0.3)	0	0	2 (0.6)
	Missing	0	0	0	0	0	0	0
	Total	255 (76.1)	61 (18.2)	12 (3.6)	4 (1.2)	3 (0.9)	0	335 (100.0)
Tirzepatide MTD group continuing treatment at Week 36 (*N* = 335)	Not at all limited	120 (35.8)	12 (3.6)	3 (0.9)	1 (0.3)	0	0	136 (40.6)
	A little limited	85 (25.4)	28 (8.4)	3 (0.9)	0	1 (0.3)	0	117 (34.9)
	Moderately limited	36 (10.7)	19 (5.7)	3 (0.9)	0	0	0	58 (17.3)
	Very much limited	11 (3.3)	8 (2.4)	2 (0.6)	0	0	1 (0.3)	22 (6.6)
	Extremely limited	2 (0.6)	0	0	0	0	0	2 (0.6)
	Missing	0	0	0	0	0	0	0
	Total	254 (75.8)	67 (20.0)	11 (3.3)	1 (0.3)	1 (0.3)	1 (0.3)	335 (100.0)

Double‐blind treatment period (Weeks 36–88)—Efficacy analysis set								
		Response group at Week 88,^**a**^ *n* (%)						
Treatment	Response group at Week 36, *n* (%)	Not at all limited	A little limited	Moderately limited	Very much limited	Extremely limited	Missing	Total
Placebo (*N* = 335)	Not at all limited	147 (43.9)	47 (14.0)	10 (3.0)	4 (1.2)	0	45 (13.4)	253 (75.5)
	A little limited	19 (5.7)	25 (7.5)	2 (0.6)	1 (0.3)	0	14 (4.2)	61 (18.2)
	Moderately limited	0	3 (0.9)	3 (0.9)	3 (0.9)	0	3 (0.9)	12 (3.6)
	Very much limited	0	1 (0.3)	0	1 (0.3)	0	2 (0.6)	4 (1.2)
	Extremely limited	1 (0.3)	2 (0.6)	0	0	0	0	3 (0.9)
	Missing	1 (0.3)	0	1 (0.3)	0	0	0	2 (0.6)
	Total	168 (50.1)	78 (23.3)	16 (4.8)	9 (2.7)	0	64 (19.1)	335 (100.0)
Tirzepatide MTD (*N* = 335)	Not at all limited	201 (60.0)	14 (4.2)	8 (2.4)	1 (0.3)	1 (0.3)	28 (8.4)	253 (75.5)
	A little limited	33 (9.9)	21 (6.3)	4 (1.2)	0	0	9 (2.7)	67 (20.0)
	Moderately limited	3 (0.9)	2 (0.6)	2 (0.6)	3 (0.9)	0	1 (0.3)	11 (3.3)
	Very much limited	1 (0.3)	0	0	0	0	0	1 (0.3)
	Extremely limited	0	1 (0.3)	0	0	0	0	1 (0.3)
	Missing	2 (0.6)	0	0	0	0	0	2 (0.6)
	Total	240 (71.6)	38 (11.3)	14 (4.2)	4 (1.2)	1 (0.3)	38 (11.3)	335 (100.0)

Entire study period (Weeks 0–88)—Randomized population—Excluding data after study drug discontinuation								
		Response group at Week 88, *n* (%)						
Treatment	Response group at Week 0, *n* (%)	Not at all limited	A little limited	Moderately limited	Very much limited	Extremely limited	Missing	Total
Tirzepatide MTD group randomized to placebo at Week 36 (*N* = 335)	Not at all limited	94 (28.1)	19 (5.7)	1 (0.3)	1 (0.3)	0	25 (7.5)	140 (41.8)
	A little limited	47 (14.0)	35 (10.4)	6 (1.8)	2 (0.6)	0	17 (5.1)	107 (31.9)
	Moderately limited	21 (6.3)	20 (6.0)	7 (2.1)	2 (0.6)	0	18 (5.4)	68 (20.3)
	Very much limited	5 (1.5)	4 (1.2)	2 (0.6)	4 (1.2)	0	3 (0.9)	18 (5.4)
	Extremely limited	1 (0.3)	0	0	0	0	1 (0.3)	2 (0.6)
	Missing	0	0	0	0	0	0	0
	Total	168 (50.1)	78 (23.3)	16 (4.8)	9 (2.7)	0	64 (19.1)	335 (100.0)
Tirzepatide MTD group continuing treatment at Week 36 (*N* = 335)	Not at all limited	114 (34.0)	9 (2.7)	0	1 (0.3)	1 (0.3)	11 (3.3)	136 (40.6)
	A little limited	77 (23.0)	11 (3.3)	9 (2.7)	2 (0.6)	0	18 (5.4)	117 (34.9)
	Moderately limited	35 (10.4)	13 (3.9)	3 (0.9)	1 (0.3)	0	6 (1.8)	58 (17.3)
	Very much limited	12 (3.6)	5 (1.5)	2 (0.6)	0	0	3 (0.9)	22 (6.6)
	Extremely limited	2 (0.6)	0	0	0	0	0	2 (0.6)
	Missing	0	0	0	0	0	0	0
	Total	240 (71.6)	38 (11.3)	14 (4.2)	4 (1.2)	1 (0.3)	38 (11.3)	335 (100.0)

Abbreviations: MTD, maximum tolerated dose; n, number of participants in the specified category; N, total number of participants in the treatment group.

^a^ The post‐baseline result used the Week 88 value excluding the off‐treatment data.

During the entire study period, the proportion of participants who reported that their physical activity was “not at all limited” substantially increased from Weeks 0 to 88 in the tirzepatide MTD group (Week 0: 40.6% to Week 88: 71.6%), while it was similar in the placebo group (41.8%–50.1%) (Table 2).

### 3.3 Post Hoc Analyses

#### 3.3.1 Association of Weight Reduction With PROs in the Tirzepatide Group

Tirzepatide‐treated participants who achieved greater weight reduction (≥ 5% to ≥ 30%) at Week 88 showed greater improvements in SF‐36v2 PCS, MCS, and domain (except Mental Health for which improvement was similar across weight reduction categories) scores (Figure S1A); IWQOL‐Lite‐CT Total and composite scores (Figure S2A); and EQ VAS scores during the tirzepatide lead‐in period (Weeks 0–36) (Table S2). These improvements in PROs were sustained across weight reduction categories from Weeks 36 to 88 (Figures S1B, S2B, and Table S2).

During the study period (Weeks 0 to 88), a greater weight reduction (≥ 5%–≥ 30%) with tirzepatide was associated with increased mean improvements in the SF‐36v2 PCS (range: 6.2 for ≥ 5% to 7.6 for ≥ 30%) and most domain scores (Physical Functioning: 6.6–7.8; Role‐Physical: 4.4–5.2; Bodily Pain: 4.3–5.8; General Health: 6.3–6.7; Vitality: 4.5–5.7; Social Functioning: 2.3–2.9; Mental Health: 2.4–3.0) (Figure S1C). Similarly, a greater weight reduction was associated with greater mean improvements from baseline in IWQOL‐Lite‐CT Total (range: 28.5 for ≥ 5% to 33.1 for ≥ 30%) and composite (Physical Function: 26.6–30.8; Physical: 24.9–29.2; Psychosocial: 30.4–35.3) scores (Figure S2C), and EQ VAS scores (13.8–16.2) (Table S2).

#### 3.3.2 Association of Limitations in Physical Activity at Baseline With PROs in the Tirzepatide Group

For tirzepatide lead‐in period (Weeks 0–36), mean improvements in SF‐36v2 PCS, MCS, and domain scores; IWQOL‐Lite‐CT Total and composite scores; and EQ‐5D‐5L scores were numerically greater in tirzepatide‐treated participants with limitations in physical functioning at baseline than in those without limitations (Table 3). These improvements in PROs were sustained from Weeks 36 to 88 in both groups (Table 3).

**TABLE 3:** Mean change in baseline Patient Global Impression of Status for physical activity among tirzepatide‐treated participants.

Patient‐reported outcomes score, mean (SD)	Baseline Patient Global Impression of Status for physical activity response category	
	Participants without physical function limitations^a^	Participants with physical function limitations^b^
Tirzepatide lead‐in period (Weeks 0–36)—Randomized population		
Short Form‐36 Version 2 Health Survey Acute Form (SF‐36v2)^c^ scores (general quality of life survey)	*n* = 252	*n* = 82
Physical Component Summary	4.3 (5.46)	11.3 (7.39)
Mental Component Summary	0.6 (5.78)	3.2 (9.69)
Domain scores		
Physical Functioning	4.3 (6.30)	10.7 (8.29)
Role‐Physical	3.2 (5.80)	8.5 (8.75)
Bodily Pain	2.1 (7.68)	8.7 (8.84)
General Health	4.7 (6.39)	10.4 (7.27)
Vitality	3.1 (6.55)	9.1 (9.49)
Social Functioning	1.4 (6.07)	5.5 (10.27)
Role‐Emotional	1.4 (6.93)	5.0 (11.95)
Mental Health	1.1 (6.55)	4.0 (8.16)
Impact of Weight on Quality of Life‐Lite‐Clinical Trials Version^d^ (IWQOL‐Lite‐CT; weight‐related quality of life questionnaire)	*n* = 252	*n* = 82
Total	19.3 (16.65)	37.1 (21.59)
Physical Function composite	17.8 (19.76)	35.5 (23.88)
Physical composite	16.6 (17.76)	34.4 (22.13)
Psychosocial composite	20.8 (18.96)	38.6 (24.0)
EQ‐5D‐5L (health status questionnaire)	*n* = 241	*n* = 79
Health State Index (UK)	0 (0.13)	0.2 (0.18)
EQ Visual Analogue Scale	9.3 (15.02)	20.9 (17.79)
Double‐blind treatment period (Weeks 36–88)—Efficacy analysis set		
SF‐36v2^c^	*n* = 229	*n* = 74
Physical Component Summary	0.2 (4.82)	0.8 (4.76)
Mental Component Summary	0 (5.57)	0.4 (7.94)
Domain scores		
Physical Functioning	0.3 (5.00)	2.4 (4.24)
Role‐Physical	−0.3 (4.38)	0.5 (5.73)
Bodily Pain	0.6 (6.84)	−0.3 (7.12)
General Health	0.5 (5.59)	−0.3 (6.33)
Vitality	−0.5 (6.35)	0.9 (7.54)
Social Functioning	−0.4 (5.86)	0.5 (7.28)
Role‐Emotional	0.3 (5.34)	1.0 (10.33)
Mental Health	0.4 (6.36)	0.4 (6.78)
IWQOL‐Lite‐CT^d^	*n* = 227	*n* = 74
Total	4.4 (10.72)	6.0 (13.82)
Physical Function composite	4.3 (14.15)	5.2 (16.60)
Physical composite	3.7 (12.52)	4.9 (15.07)
Psychosocial composite	4.7 (11.95)	6.6 (15.38)
EQ‐5D‐5L	*n* = 227	*n* = 72
Health State Index (UK)	0 (0.12)	0 (0.17)
EQ Visual Analogue Scale	0.9 (10.07)	2.0 (9.64)
Entire study period (Weeks 0–88)—Randomized population—Excluding data after study drug discontinuation		
SF‐36v2^c^	*n* = 230	*n* = 74
Physical Component Summary	4.5 (6.12)	11.6 (7.44)
Mental Component Summary	0.9 (6.23)	3.8 (10.38)
Domain scores		
Physical Functioning	4.6 (6.66)	12.7 (8.40)
Role‐Physical	3.0 (6.20)	8.6 (9.64)
Bodily Pain	2.8 (8.72)	8.7 (9.15)
General Health	5.2 (6.39)	9.7 (8.61)
Vitality	2.8 (7.32)	9.5 (9.38)
Social Functioning	1.1 (7.15)	5.9 (10.11)
Role‐Emotional	2.1 (6.83)	6.0 (12.33)
Mental Health	1.7 (6.90)	4.8 (9.17)
IWQOL‐Lite‐CT^d^	*n* = 228	*n* = 74
Total score	23.9 (17.63)	42.0 (21.38)
Physical Function composite	22.1 (20.59)	40.1 (23.96)
Physical composite	20.3 (18.55)	38.7 (23.27)
Psychosocial composite	25.8 (19.89)	43.8 (23.33)
EQ‐5D‐5L	*n* = 221	*n* = 71
Health State Index (UK)	0.0 (0.15)	0.2 (0.19)
EQ Visual Analogue Scale	10.5 (15.63)	23.5 (17.23)

*Note*: Data are presented as mean change from baseline (Week 0) or randomization (Week 36) to Week 88 in patient‐reported outcomes using last observation carried forward.

Abbreviation: *n*, number of participants with non‐missing baseline and at least 1 non‐missing post‐baseline value.

^a^ “Not at all limited” or “a little limited” physical function at baseline.

^b^ “Moderately limited,” or “very much limited,” or “extremely limited” physical function at baseline.

^c^ The SF‐36v2 scores are norm‐based scores, i.e., scores transformed to a scale in which the 2009 US general population has a mean score of 50 and an SD of 10. A higher score indicates better health.

^d^ Scores are transformed to a scale of 0 to 100, with higher scores reflecting better levels of functioning.

Throughout the study (Weeks 0–88) mean improvements in SF‐36v2 scores were greater in participants with versus without limitations in physical functioning at baseline: PCS (11.6 vs. 4.5), MCS (3.8 vs. 0.9), and domain (Physical Functioning: 12.7 vs. 4.6; Role‐Physical: 8.6 vs. 3.0; Bodily Pain: 8.7 vs. 2.8; General Health: 9.7 vs. 5.2; Vitality: 9.5 vs. 2.8; Social Functioning: 5.9 vs. 1.1; Role‐Emotional: 6.0 vs. 2.1; Mental Health 4.8 vs. 1.7) scores. Similarly, mean improvements were numerically greater for IWQOL‐Lite‐CT Total (42.0 vs. 23.9) and composite scores (Physical Function: 40.1 vs. 22.1; Physical: 38.7 vs. 20.3; and Psychosocial: 43.8 vs. 25.8), and EQ VAS scores (23.5 vs. 10.5) (Table 3).

#### 3.3.3 Association of Weight Regain With PROs Among Participants in the Placebo Group

During Weeks 36 to 88, greater weight regain among participants who switched to placebo was associated with worsening of SF‐36v2 PCS (mean range: −0.9 for < 25% to −3.4 for ≥ 75% weight regain), MCS (−0.7 to −3.6), and domain scores (Physical Functioning: −2.1 to −3.7; Role‐Physical: −0.4 to −2.7; Bodily Pain: 0.1 to −3.9; General Health: −0.3 to −4.5; Vitality: −1.6 to −4.4; Social Functioning: −0.9 to −2.1; Role‐Emotional: −0.2 to −2.4; and Mental Health: −1.1 to −5.3). Similarly, worsening was also seen in IWQOL‐Lite‐CT Total (mean range: −0.8 for < 25% to −12.8 for ≥ 75% weight regain) and composite (Physical Function: −0.9 to −11.8; Physical: −0.9 to −3.4; and Psychosocial: −0.3 to −13.5) scores, and EQ VAS scores (−2.0 to −6.6) (Table S3).

## 4 Discussion

Participants in the SURMOUNT‐4 trial who continued to receive tirzepatide MTD experienced a significant and sustained improvement from baseline in all domains of general health‐related (SF‐36v2 and EQ‐5D‐5L) and weight‐related (IWQOL‐Lite‐CT) quality of life when compared to participants who switched to placebo. The number of participants who achieved clinically meaningful improvements in physical functioning was also higher in the tirzepatide MTD than in the placebo group. Consistent with other phase 3 studies [23, 24, 25, 26], greater weight reduction among tirzepatide‐treated participants was associated with greater improvements in quality of life. Tirzepatide‐treated participants who reported limitations in physical functioning at baseline showed greater improvement in PROs than those who did not report these limitations. Participants who regained weight after switching to placebo showed worsening of PROs; greater weight regain was associated with greater worsening of PROs.

In addition to the SURMOUNT‐4 trial [12], several other withdrawal studies evaluating the efficacy of obesity medications have shown that participants regain substantial weight after withdrawal of pharmacotherapy [27, 28, 29, 30]. In STEP 4, HRQoL worsened in participants who switched to placebo after receiving semaglutide 2.4 mg during the run‐in period compared to those who continued treatment with semaglutide [23]. Consistent with these findings, improvements in PROs observed during the tirzepatide lead‐in period (Weeks 0 to 36) in the current study were sustained during the double‐blind period (Weeks 36 to 88) in participants who were maintained on tirzepatide. However, improvements in PROs were lost in participants who switched to placebo, suggesting that the benefits observed with tirzepatide MTD may be reversible after its withdrawal. Given the chronic and relapsing nature of obesity, the study findings underscore the importance of continuing pharmacotherapy to maintain the weight reduction and HRQoL benefits in people with obesity.

The IWQOL‐Lite‐CT has been developed in accordance with the FDA's PRO guidance to specifically assess weight‐related functioning in clinical trials [15]. The baseline IWQOL‐Lite‐CT scores (58.1–59.6) were low in the current study. In a confirmatory psychometric analysis, a within‐participant mean change of 16.6 points for IWQOL‐Lite‐CT Total score and 13.5–16.2 points for IWQOL‐Lite‐CT composite scores was considered meaningful in people with obesity or overweight [16]. During the study period, these clinically meaningful thresholds were not only achieved in both treatment groups, but also were significantly greater with tirzepatide MTD versus placebo for the IWQOL‐Lite‐CT Total (27.8 vs. 16.7) and composite scores (24.4–29.6 vs. 15.3–17.5). At Week 88, treatment with tirzepatide was associated with significant improvements in SF‐36v2 PCS, MCS, and domain scores and in EQ‐VAS scores, compared with placebo (*p* < 0.05, for all). These findings are in congruence with the available literature, where treatment with tirzepatide was associated with significant improvements in SF36 v2 scores and EQ‐VAS scores (*p* < 0.05, for all) compared with placebo [31, 32].

In a qualitative study examining meaningful change in physical function associated with weight reduction, nearly two‐thirds of people with overweight or obesity considered a 5% weight reduction sufficient to yield some benefit in physical functioning. However, all participants considered ≥ 10% body weight reduction necessary for a meaningful and noticeable improvement in their physical functioning [33]. At Week 88, tirzepatide‐treated participants with ≥ 5% body weight reduction showed meaningful improvements in SF‐36v2 Physical Functioning domain score (mean: 6.6) and IWQOL‐Lite‐CT Physical Function composite score (mean: 26.6). These results suggest that improvements in HRQoL and physical functioning could be due to weight reduction.

A patient‐centric approach and partnership between the patient and clinician is needed to manage obesity [34]. While weight and BMI are widely used to assess the efficacy of obesity medications, it is important to integrate outcomes meaningful to patients in treatment decisions and to provide a holistic view of patients' health [35]. This study evaluated the effect of continuing treatment with tirzepatide MTD versus switching to placebo on various PROs assessing health‐related and weight‐related quality of life across physical and mental health domains. Our study findings are consistent with those of other phase 3 SURMOUNT studies that have shown improvement in quality of life with tirzepatide versus placebo [25, 26, 36, 37, 38, 39, 40].

This study assessed PROs in a large patient population with obesity or overweight. The randomized withdrawal design and the duration of the open‐label lead‐in period allowed the study to assess the maintenance of HRQoL. The dose escalation strategies implemented during the open‐label lead‐in period helped to maximize tolerability and may assist prescribers in optimizing treatment plans for obesity management.

The study design did not allow for dose adjustments after randomization and did not evaluate the effects of intensive behavioral therapy on maintaining body weight reduction. Participants who tolerated initial treatment with tirzepatide MTD (10 or 15 mg) may represent a subgroup of the general population. The proportion of participants with physical function limitations at baseline was relatively low. Future studies should focus on people with severe baseline physical function limitations to understand the effectiveness of tirzepatide in real‐world clinical settings. The post hoc analyses were descriptive among tirzepatide‐treated participants; no comparison between tirzepatide and placebo groups was performed due to the lack of corresponding data for the placebo group.

## 5 Conclusion

In the SURMOUNT‐4 trial in participants with obesity or overweight, continued treatment with tirzepatide was associated with maintaining improvements in HRQoL, while treatment withdrawal was associated with worsening of HRQoL. Limitations in physical function at baseline and greater weight reduction among tirzepatide‐treated participants were associated with numerically greater improvements in HRQoL. Greater weight regain after switching to placebo was associated with worsening of HRQoL.

## Author Contributions

**Theresa Hunter Gibble:** conception and design of the work, interpretation of data for the work, and critical review of the work for important intellectual content. **Dachuang Cao:** conception and design of the work, acquisition of data for the work, analysis of data for the work, interpretation of data for the work, and critical review of the work for important intellectual content. **Madhumita Murphy:** analysis of data for the work, interpretation of data for the work, drafting of the work, and critical review of the work for important intellectual content. **Irina Jouravskaya, Birong Liao**, and **Harold Edward Bays:** interpretation of data for the work and critical review of the work for important intellectual content.

## Conflicts of Interest

Theresa Hunter Gibble, Dachuang Cao, Madhumita Murphy, Irina Jouravskaya, and Birong Liao are employees and stockholders of Eli Lilly and Company, Indianapolis, United States. Harold Edward Bays (H.E.B.)'s research site institution has received research grants from 89Bio, Allergan, Alon Medtech/Epitomee, Aligos, Altimmune, Amgen, Anji Pharma, AbbVie, AstraZeneca, Bioage, Bionime, Boehringer Ingelheim, Carmot, Chorus/Bioage, Eli Lilly, Esperion, Evidera, Fractyl, GlaxoSmithKline, HighTide, Home Access, Horizon, Ionis, Kallyope, LG‐Chem, Madrigal, Merck, Mineralys, New Amsterdam, Novartis, Novo Nordisk, Pfizer, Regeneron, Satsuma, Selecta, Shionogi, Skye/Birdrock, TIMI, Veru, Viking, and Vivus. H.E.B. has served as a consultant/advisor for 89Bio, Altimmune, Amgen, Boehringer Ingelheim, Kiniksa, HighTide, Lilly, Novo Nordisk, Regeneron, Veru, Zomagen, and ZyVersa.

## Supporting information


**Data S1:** Supplementary Tables.

## Data Availability

Lilly provides access to all individual participant data collected during the trial, after anonymization, with the exception of pharmacokinetic or genetic data. Data are available to request 6 months after the indication studied has been approved in the US and EU and after primary publication acceptance, whichever is later. No expiration date of data requests is currently set once data are made available. Access is provided after a proposal has been approved by an independent review committee identified for this purpose and after receipt of a signed data sharing agreement. Data and documents, including the study protocol, statistical analysis plan, clinical study report, and blank or annotated case report forms, will be provided in a secure data sharing environment. For details on submitting a request, see the instructions provided at https://www.vivli.org.

## References

[1] “Health Risks of Overweight & Obesity,” NIDDK, https://www.niddk.nih.gov/health‐information/weight‐management/adult‐overweight‐obesity/health‐risks.

[2] H. Rozjabek , J. Fastenau , A. LaPrade , and N. Sternbach , “Adult Obesity and Health‐Related Quality of Life, Patient Activation, Work Productivity, and Weight Loss Behaviors in the United States,” Diabetes, Metabolic Syndrome and Obesity 13 (2020): 2049–2055. 10.2147/DMSO.S245486 PMC7306452 32606863

[3] K. Barnard‐Kelly , T. Battelino , F. C. Brosius , et al., “Defining Patient‐Reported Outcomes in Diabetes, Obesity, Cardiovascular Disease, and Chronic Kidney Disease for Clinical Practice Guidelines‐Perspectives of the Taskforce of the Guideline Workshop,” Cardiovascular Diabetology 24 (2025): 68. 39920737 10.1186/s12933-024-02550-2 PMC11806799

[4] D. B. Sarwer and H. M. Polonsky , “The Psychosocial Burden of Obesity,” Endocrinology and Metabolism Clinics 45, no. 3 (2016): 677–688. 27519139 10.1016/j.ecl.2016.04.016 PMC6052856

[5] N. A. ElSayed , G. Aleppo , V. R. Aroda , et al., “8. Obesity and Weight Management for the Prevention and Treatment of Type 2 Diabetes: Standards of Care in Diabetes—2023,” Diabetes Care 46, no. S1 (2023): S128–S139. 36507637 10.2337/dc23-S008 PMC9810466

[6] W. T. Garvey , J. I. Mechanick , E. M. Brett , et al., “American Association of Clinical Endocrinologists and American College of Endocrinology Comprehensive Clinical Practice Guidelines for Medical Care of Patients With Obesity,” Endocrine Practice 22, no. S3 (2016): 1–203. 10.4158/EP161365.GL 27219496

[7] B. G. Tchang , M. Aras , A. Wu , L. J. Aronne , and A. P. Shukla , “Long‐Term Weight Loss Maintenance With Obesity Pharmacotherapy: A Retrospective Cohort Study,” Obesity Science & Practice 8, no. 3 (2022): 320–327. 35664243 10.1002/osp4.575 PMC9159566

[8] M. L. Butryn , V. Webb , and T. A. Wadden , “Behavioral Treatment of Obesity,” Psychiatric Clinics of North America 34, no. 4 (2011): 841–859. 22098808 10.1016/j.psc.2011.08.006 PMC3233993

[9] R. L. Kolotkin and J. R. Andersen , “A Systematic Review of Reviews: Exploring the Relationship Between Obesity, Weight Loss and Health‐Related Quality of Life,” Clinical Obesity 7, no. 5 (2017): 273–289. 28695722 10.1111/cob.12203 PMC5600094

[10] “FDA Approves New Medication for Chronic Weight Management,” FDA, published November 8, 2023, https://www.fda.gov/news‐events/press‐announcements/fda‐approves‐new‐medication‐chronic‐weight‐management.

[11] “Novel Drug Approvals for 2022,” FDA, https://www.fda.gov/drugs/novel‐drug‐approvals‐fda/novel‐drug‐approvals‐2022.

[12] L. J. Aronne , N. Sattar , D. B. Horn , et al., “Continued Treatment With Tirzepatide for Maintenance of Weight Reduction in Adults With Obesity: The SURMOUNT‐4 Randomized Clinical Trial,” JAMA 331, no. 1 (2024): 38–48. 38078870 10.1001/jama.2023.24945 PMC10714284

[13] M. Maruish , User's Manual for the SF‐36v2 Health Survey (Quality Metric Incorporated, 2011).

[14] K. W. Wyrwich , W. M. Tierney , A. N. Babu , K. Kroenke , and F. D. Wolinsky , “A Comparison of Clinically Important Differences in Health‐Related Quality of Life for Patients With Chronic Lung Disease, Asthma, or Heart Disease,” Health Services Research 40, no. 2 (2005): 577–592. 15762908 10.1111/j.1475-6773.2005.00373.x PMC1361158

[15] R. L. Kolotkin , V. S. Williams , C. M. Ervin , et al., “Validation of a New Measure of Quality of Life in Obesity Trials: Impact of Weight on Quality of Life‐Lite Clinical Trials Version,” Clinical Obesity 9, no. 3 (2019): e12310. 30993900 10.1111/cob.12310 PMC6593657

[16] R. L. Kolotkin , V. S. L. Williams , L. von Huth Smith , et al., “Confirmatory Psychometric Evaluations of the Impact of Weight on Quality of Life‐Lite Clinical Trials Version (IWQOL‐Lite‐CT),” Clinical Obesity 11, no. 5 (2021): e12477, 10.1111/cob.12477. 34296522 PMC9285468

[17] EuroQol Research Foundation , EQ‐5D‐5L User Guide, Version 3.0 (EuroQol, 2021), https://euroqol.org/information‐and‐support/documentation/user‐guides/.

[18] P. Dolan , “Modeling Valuations for EuroQol Health States,” Medical Care 35, no. 11 (1997): 1095–1108. 9366889 10.1097/00005650-199711000-00002

[19] N. Luo , J. A. Johnson , and S. J. Coons , “Using Instrument‐Defined Health State Transitions to Estimate Minimally Important Differences for Four Preference‐Based Health‐Related Quality of Life Instruments,” Medical Care 48, no. 4 (2010): 365–371. 20355266 10.1097/mlr.0b013e3181c162a2

[20] L. M. Warkentin , S. R. Majumdar , J. A. Johnson , et al., “Weight Loss Required by the Severely Obese to Achieve Clinically Important Differences in Health‐Related Quality of Life: Two‐Year Prospective Cohort Study,” BMC Medicine 12 (2014): 175, 10.1186/s12916-014-0175-5. 25315502 PMC4212133

[21] J. M. Fermont , J. M. Blazeby , C. A. Rogers , S. Wordsworth , and Group B‐B‐SSM , “The EQ‐5D‐5L Is a Valid Approach to Measure Health Related Quality of Life in Patients Undergoing Bariatric Surgery,” PLoS One 12, no. 12 (2017): e0189190. 29252996 10.1371/journal.pone.0189190 PMC5734736

[22] G. H. Guyatt , D. Osoba , A. W. Wu , K. W. Wyrwich , G. R. Norman , and Clinical Significance Consensus Meeting Group , “Methods to Explain the Clinical Significance of Health Status Measures,” Mayo Clinic Proceedings 77 (2002): 371–383. 11936935 10.4065/77.4.371

[23] D. Rubino , J. B. Bjorner , N. Rathor , et al., “Effect of Semaglutide 2.4 Mg on Physical Functioning and Weight‐ and Health‐Related Quality of Life in Adults With Overweight or Obesity: Patient‐Reported Outcomes From the STEP 1‐4 Trials,” Diabetes, Obesity & Metabolism 26, no. 7 (2024): 2945–2955, 10.1111/dom.15620. 38698650

[24] R. L. Kolotkin , K. Fujioka , M. L. Wolden , J. H. Brett , and J. B. Bjorner , “Improvements in Health‐Related Quality of Life With Liraglutide 3.0 Mg Compared With Placebo in Weight Management,” Clinical Obesity 6, no. 4 (2016): 233–242, 10.1111/cob.12146. 27198973 PMC5084798

[25] T. Hunter Gibble , D. Cao , M. X. Zhang , N. Agarwal Xavier , and J. Ling Poon , “1700‐P: Weight Reduction Is Associated With Improved Quality of Life in Participants With Obesity or Overweight and Type 2 Diabetes—Results From Phase 3 SURMOUNT‐2 Trial,” Diabetes 73, no. S1 (2024): 1700‐P, 10.2337/db24-1700-P. PMC12006608 40120035

[26] T. Hunter , T. Gibble , D. Cao , et al., “#1704946 Improvements in Physical Functioning Among Participants With Obesity With Limitations in Physical Function at Baseline: Results From SURMOUNT‐1 and SURMOUNT‐2 Trials,” Endocrine Practice 30, no. 5 (2024): S70, 10.1016/j.eprac.2024.03.262.

[27] D. Rubino , N. Abrahamsson , M. Davies , et al., “Effect of Continued Weekly Subcutaneous Semaglutide vs Placebo on Weight Loss Maintenance in Adults With Overweight or Obesity: The STEP 4 Randomized Clinical Trial,” JAMA 325, no. 14 (2021): 1414–1425, 10.1001/jama.2021.3224. 33755728 PMC7988425

[28] L. Sjöström , A. Rissanen , T. Andersen , et al., “Randomised Placebo‐Controlled Trial of Orlistat for Weight Loss and Prevention of Weight Regain in Obese Patients. European Multicentre Orlistat Study Group,” Lancet 352, no. 9123 (1998): 167–172, 10.1016/s0140-6736(97)11509-4. 9683204

[29] S. R. Smith , N. J. Weissman , C. M. Anderson , et al., “Multicenter, Placebo‐Controlled Trial of Lorcaserin for Weight Management,” New England Journal of Medicine 363, no. 3 (2010): 245–256, 10.1056/NEJMoa0909809. 20647200

[30] W. P. James , A. Astrup , N. Finer , et al., “Effect of Sibutramine on Weight Maintenance After Weight Loss: A Randomised Trial,” Lancet 356, no. 9248 (2000): 2119–2125, 10.1016/s0140-6736(00)03491-7. 11191537

[31] K. A. Gudzune , A. Stefanski , D. Cao , et al., “Association Between Weight Reduction Achieved With Tirzepatide and Quality of Life in Adults With Obesity: Results From the SURMOUNT‐1 Study,” Diabetes, Obesity and Metabolism 27, no. 2 (2025): 539–550. 10.1111/dom.16046 PMC11701187 39497468

[32] T. Hunter Gibble , D. Cao , X. M. Zhang , N. A. Xavier , J. L. Poon , and A. Fitch , “Tirzepatide Was Associated With Improved Health‐Related Quality of Life in Adults With Obesity or Overweight and Type 2 Diabetes: Results From the Phase 3 SURMOUNT‐2 Trial,” Diabetes Therapy 16, no. 5 (2025): 977–991. 40120035 10.1007/s13300-025-01723-w PMC12006608

[33] J. L. Poon , C. Marshall , C. Johnson , et al., “A Qualitative Study to Examine Meaningful Change in Physical Function Associated With Weight‐Loss,” Quality of Life Research 32, no. 5 (2023): 1329–1340, 10.1007/s11136-022-03191-2. 35867321 PMC9305034

[34] M. A. Cornier , “A Review of Current Guidelines for the Treatment of Obesity,” American Journal of Managed Care 28, no. 15 Suppl (2022): S288–S296, 10.37765/ajmc.2022.89292. 36525676

[35] E. Ciemins , V. Joshi , D. Horn , J. Nadglowski , A. Ramasamy , and J. Cuddeback , “Measuring What Matters: Beyond Quality Performance Measures in Caring for Adults With Obesity,” Population Health Management 24, no. 4 (2021): 482–491, 10.1089/pop.2020.0109. 33180000 PMC8403197

[36] J. L. C. D. Poon , I. Jouravskaya , F. Wang , B. Falcon , and J. Simonetti , “Weight Reduction Is Associated With Improved Quality of Life in Patients With Obesity: SURMOUNT‐1,” Obesity 31, no. S2 (2023): 183, 10.1002/oby.23939.

[37] J. L. Poon , S. Zhang , H. Kan , et al., “OR10‐05 Improved Mental and Psychosocial Patient‐Reported Outcomes Among People With Obesity Treated With Tirzepatide: Results From SURMOUNT‐1 Study,” Journal of the Endocrine Society 7, no. S1 (2023): bvad114.011, 10.1210/jendso/bvad114.011.

[38] T. Hunter , T. H. Gibble , D. Cao , X. M. Zhang , N. A. Xavier , and J. L. Poon , “#1704957 Tirzepatide Improves Health‐Related Quality of Life in Participants With Obesity or Overweight and Type 2 Diabetes: Results From Phase 3 SURMOUNT‐2 Trial,” Endocrine Practice 30, no. 5 (2024): S70, 10.1016/j.eprac.2024.03.263.

[39] T. Hunter , T. H. Gibble , D. Cao , T. Forrester , and J. F. Brumm , “#1702836 Tirzepatide Improved Health‐Related Quality of Life in Adults With Obesity or Overweight: Results From the SURMOUNT‐3 Phase 3 Trial,” Endocrine Practice 30, no. 5 (2024): S69, 10.1016/j.eprac.2024.03.260.

[40] T. Hunter Gibble , D. Cao , T. D. Forrester , J. Fraseur Brumm , and A. Chao , “1676‐P: Tirzepatide Improved Mental and Psychosocial Function in Adults With Obesity or Overweight in the SURMOUNT‐3 Trial,” Diabetes 73, no. S1 (2024): 1676‐P, 10.2337/db24-1676-P.

