# Supplementary material for: Tirzepatide Associated With Improved Health‐Related Quality of Life in Adults With Obesity or Overweight in SURMOUNT‐4
Source: Obesity (Silver Spring). 2025 Sep 3;33(11):2076–92. doi: 10.1002/oby.70011 (PMC12559773; doi:10.1002/oby.70011)
Supplement: Supplementary file 1 — Data S1: Supplementary Tables. [file OBY-33-2076-s001.docx]

**Tirzepatide associated with improved health-related quality of life in adults with obesity or overweight in SURMOUNT-4**

Theresa Hunter Gibble, PhD^1,^*, Dachuang Cao, PhD^1^, Madhumita Murphy, MD^1^, Irina Jouravskaya, MD, PhD^1^, Birong Liao, PhD^1^, Harold Edward Bays, MD^2^

**Affiliations**

^1^Eli Lilly and Company, Indianapolis, IN, USA

^2^ Louisville Metabolic and Atherosclerosis Research Center, Louisville, KY, USA

***Corresponding author**

Theresa Hunter Gibble

Eli Lilly and Company,

893 S. Delaware Street

Indianapolis, IN, 46285, USA

e-mail: hunter_theresa_marie@lilly.com

ORCID id: 0000-0001-8412-9175

**Table S1: Pre-specified and post hoc endpoints**

| **Endpoints** | **Secondary** | **Exploratory** | **Post hoc** |
| --- | --- | --- | --- |
| Mean change in SF-36v2 summary and domain scores |  |  |  |
| Physical Functioning, Role-Physical, Role-Emotional, and Mental Health domain scores | Weeks 36 to 88  Weeks 0 to 88 |  | Weeks 0 to 36 |
| PCS, MCS, and Bodily Pain, General Health, Vitality, Social Functioning domain scores | Weeks 0 to 88 | Weeks 36 to 88 | Weeks 0 to 36 |
| Mean change in IWQOL-Lite-CT Total and composite scores |  |  |  |
| Physical Function composite score | Weeks 36 to 88  Weeks 0 to 88 |  | Weeks 0 to 36 |
| Total score, and Physical and Psychosocial composite scores |  | Weeks 36 to 88  Weeks 0 to 88 | Weeks 0 to 36 |
| Mean change in PROs in participants who had physical function limitations at baseline |  |  |  |
| SF-36v2 Physical Functioning domain score |  | Weeks 36 to 88  Weeks 0 to 88 | Weeks 0 to 36 |
| IWQOL-Lite-CT Physical Function composite score |  | Weeks 36 to 88  Weeks 0 to 88 | Weeks 0 to 36 |
| Mean change in EQ-5D-5L utility score and EQ-5D-5L Visual Analog Scale score |  | Weeks 36 to 88  Weeks 0 to 88 | Weeks 0 to 36 |
| Number and proportion of participants endorsing each PGIS response category on a 5-point scale (“not at all limited” to “extremely limited”) |  | Weeks 36 to 88  Weeks 0 to 88 | Weeks 0 to 36 |
| Proportion of Participants achieving meaningful within-participant change in SF-36v2 Physical Functioning Domain Score (≥5.76) |  |  | Weeks 0 to 36  Weeks 36 to 88  Weeks 0 to 88 |
| Mean change in SF-36v2, IWQOL-Lite-CT, and EQ-5D-5L in tirzepatide-treated participants by   1. Percentage weight reduction categories (≥5%, ≥10%, ≥15%, ≥20%, ≥25%, and ≥30%) 2. Presence or absence of physical function limitations at baseline (determined by PGIS) |  |  | Weeks 0 to 36  Weeks 36 to 88  Weeks 0 to 88 |
| Mean change in SF-36v2, IWQOL-Lite-CT, and EQ-5D-5L among placebo-treated participants by weight regain categories (<25%, 25% – <50%, 50% – <75%, and ≥75%) |  |  | Weeks 36 to 88 |

Endpoints were assessed in the randomized population for Weeks 0 to 36; efficacy analysis set for Weeks 36 to 88; randomized population – excluding data after study drug discontinuation for Weeks 0 to 88.

Abbreviations: IWQOL-Lite-CT, Impact of Weight on Quality of Life-Lite-Clinical Trials Version; MCS, Mental Component Summary; PCS, Physical Component Summary; PGIS, Patient Global Impression of Status for physical activity; PRO, patient-reported outcome; SF-36v2, Short Form-36 Version 2 Health Survey acute form.

**Table S2:** Mean change in EQ-5D-5L (health status questionnaire) by weight reduction categories at Week 88

| **Patient-reported outcomes score,  mean (SD)** | **Weight reduction categories at Week 88** | | | | | |
| --- | --- | --- | --- | --- | --- | --- |
|  | **≥5%** | **≥10%** | **≥15%** | **≥20%** | **≥25%** | **≥30%** |
| **Tirzepatide lead-in period (Weeks 0 to 36) — Randomized Population** | | | | | | |
| EQ-5D-5L (health status questionnaire) | n=319 | n=312 | n=298 | n=250 | n=207 | n=135 |
| Health State Index (UK) | 0.1 (0.15) | 0.1 (0.15) | 0.1 (0.15) | 0.1 (0.16) | 0.1 (0.16) | 0.1 (0.17) |
| EQ Visual Analog Scale | 12.3 (16.43) | 12.6 (16.44) | 12.8 (16.45) | 13.8 (16.92) | 13.6 (17.41) | 14.5 (17.13) |
| **Double-blind treatment period (Weeks 36 to 88) — Efficacy analysis set** | | | | | | |
| EQ-5D-5L | n=298 | n=291 | n=279 | n=236 | n=194 | n=128 |
| Health State Index (UK) | 0 (0.13) | 0 (0.13) | 0 (0.14) | 0 (0.13) | 0 (0.13) | 0 (0.12) |
| EQ Visual Analog Scale | 1.1 (9.98) | 1.4 (9.88) | 1.4 (9.96) | 1.5 (9.70) | 2.2 (9.92) | 1.8 (10.13) |
| **Entire study period (Weeks 0 to 88) — Randomized Population – Excluding data after study drug discontinuation** | | | | | | |
| EQ-5D-5L | n=291 | n=284 | n=273 | n=229 | n=190 | n=124 |
| Health State Index (UK) | 0.1 (0.17) | 0.1 (0.16) | 0.1 (0.17) | 0.1 (0.17) | 0.1 (0.18) | 0.1 (0.16) |
| EQ Visual Analog Scale | 13.8 (16.83) | 14.3 (16.71) | 14.4 (16.79) | 15.6 (16.78) | 16.0 (17.23) | 16.2 (17.51) |

Data are presented as mean change from baseline (Week 0) or randomization (Week 36) to Week 88 in patient-reported outcomes using last observation carried forward.

Abbreviations: n, number of participants with non-missing baseline and at least 1 non-missing post-baseline value; SD, standard deviation.

**Table S3.** Mean change from Weeks 36 to 88 in patient-reported outcomes by weight regain categories among participants in the placebo group who achieved ≥10% weight reduction at Week 36

| **PRO score, mean (SD)** | **Weight regain categories** | | | |
| --- | --- | --- | --- | --- |
|  | **<25%** | **≥25% to <50%** | **≥50% to <75%** | **≥75%** |
| Short Form-36 Version 2 Health Survey acute form^a^ (SF-36 v2) scores (general quality of life survey) | n=40 | n=67 | n=80 | n=67 |
| Physical Component Summary | -0.9 (4.37) | -0.7 (4.69) | -0.6 (5.36) | -3.4 (7.27) |
| Mental Component Summary | -0.7 (6.40) | -0.6 (7.25) | -2.4 (8.89) | -3.6 (7.88) |
| Domain scores |  |  |  |  |
| Physical Functioning | -2.1 (7.17) | -1.1 (4.76) | -0.7 (5.70) | -3.7 (7.14) |
| Role-Physical | -0.4 (3.64) | -0.4 (6.46) | -0.1 (6.15) | -2.7 (8.66) |
| Bodily Pain | 0.1 (5.49) | -1.4 (7.92) | -0.4 (8.02) | -3.9 (7.93) |
| General Health | -0.3 (5.63) | -0.2 (6.02) | -3.7 (7.12) | -4.5 (7.37) |
| Vitality | -1.6 (6.19) | -0.5 (6.18) | -1.1 (7.26) | -4.4 (7.72) |
| Social Functioning | -0.9 (5.12) | 0.3 (6.98) | -2.1 (9.23) | -2.1 (10.58) |
| Role-Emotional | -0.2 (7.33) | -1.3 (9.96) | -1.3 (8.74) | -2.4 (8.71) |
| Mental Health | -1.1 (6.34) | -0.9 (6.72) | -2.7 (9.00) | -5.3 (8.18) |
| Impact of Weight on Quality of Life-Lite-Clinical Trials Version^b^ (IWQOL-Lite-CT; weight-related quality of life questionnaire) | n=41 | n=67 | n=81 | n=67 |
| Total | -0.8 (14.33) | -1.8 (12.87) | -9.1 (16.12) | -12.8 (18.71) |
| Physical function composite | -0.9 (15.77) | 0.0 (16.76) | -6.0 (18.04) | -11.8 (21.79) |
| Physical composite | -1.7 (15.93) | -0.7 (14.96) | -5.8 (16.76) | -11.5 (21.75) |
| Psychosocial composite | -0.3 (15.36) | -2.5 (13.94) | -10.8 (18.51) | -13.5 (19.87) |
| EQ-5D-5L (health status questionnaire) | n=40 | n=67 | n=81 |  |
| Health State Index (UK) | 0.0 (0.11) | -0.0 (0.14) | -0.0 (0.20) | -0.1 (0.24)  n=65 |
| EQ Visual Analog Scale | -2.0 (7.43) | -0.3 (11.41) | -5.3 (11.68) | -6.6 (16.64)  n=67 |

^a^The SF-36v2 scores are norm-based, i.e., scores transformed to a scale in which the 2009 United States general population has a mean score of 50 and a standard deviation of 10. A decrease in score represents worsening of health status.

^b^Scores are transformed to a scale of 0 to 100, with lower scores reflecting decreased level of functioning.

Abbreviations: n, number of participants with non-missing baseline and at least 1 non-missing post-baseline value.

**Figure S1:** Mean change in Short Form-36 Version 2 Health Survey acute form (SF-36v2) by weight reduction categories at Week 88

**
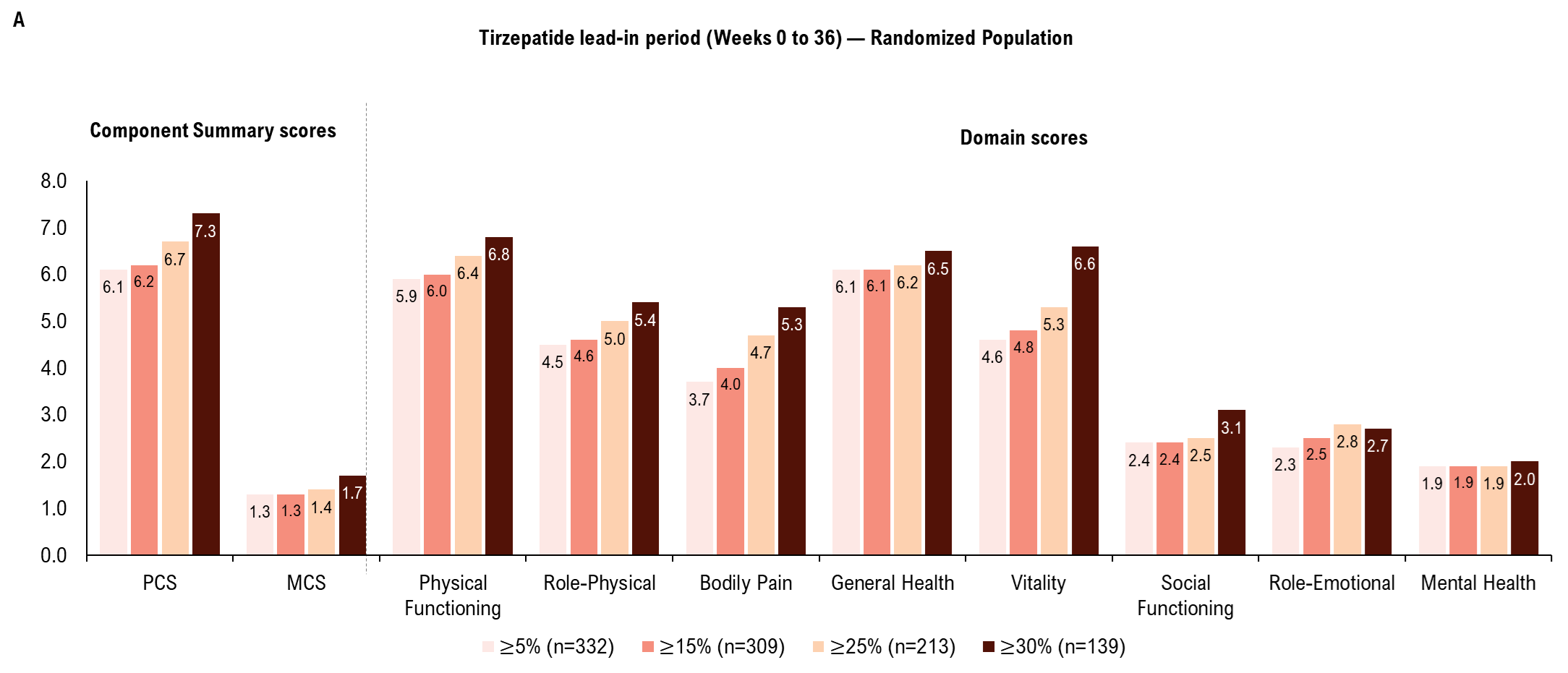
**

**
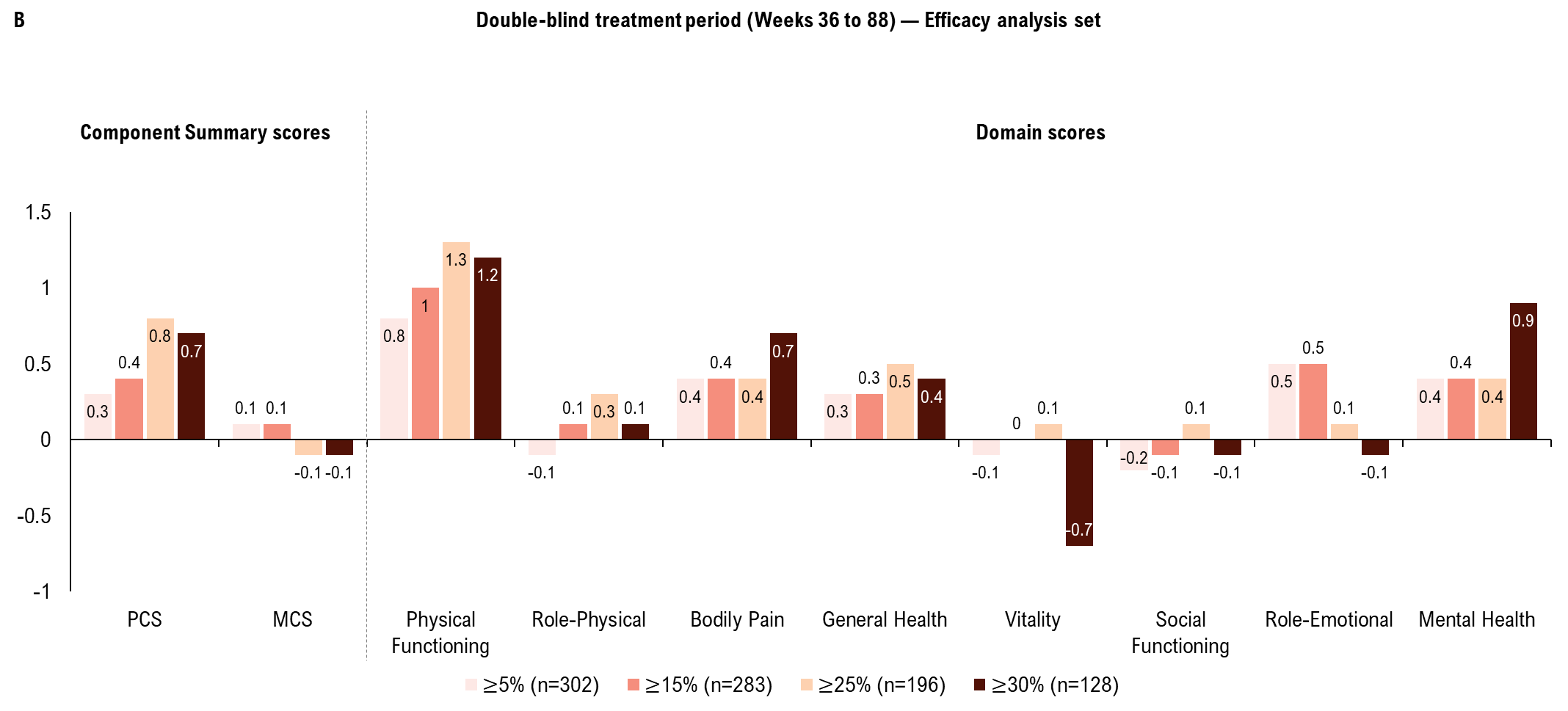
**


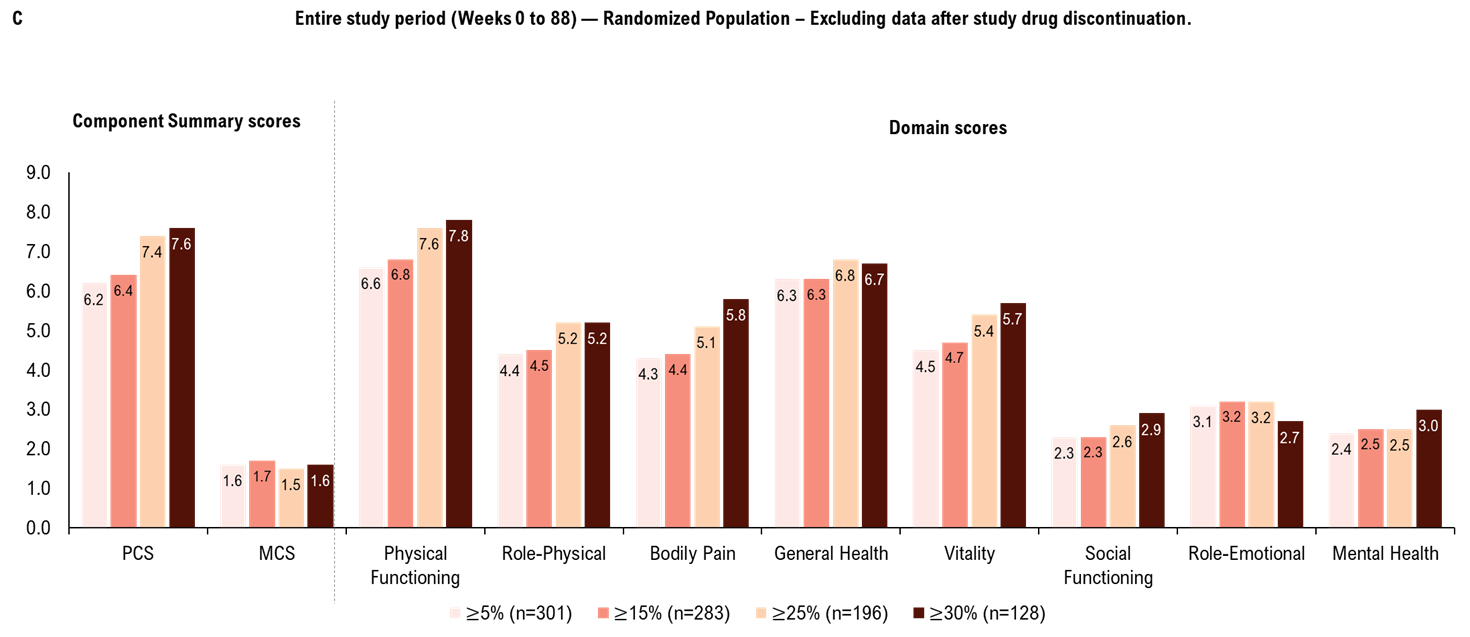


Data are presented as mean change from baseline (Week 0) or randomization (Week 36) to Week 88 in patient-reported outcomes using last observation carried forward.

The SF-36v2 scores are norm-based scores, i.e., scores transformed to a scale in which the 2009 United States general population has a mean score of 50 and an SD of 10. A higher score indicates better health.

Abbreviations: n, number of participants with non-missing baseline and at least 1 non-missing post-baseline value

**Figure S2:** Mean change in by Impact of Weight on Quality of Life-Lite-Clinical Trials Version (IWQOL-Lite-CT) by weight reduction categories at Week 88


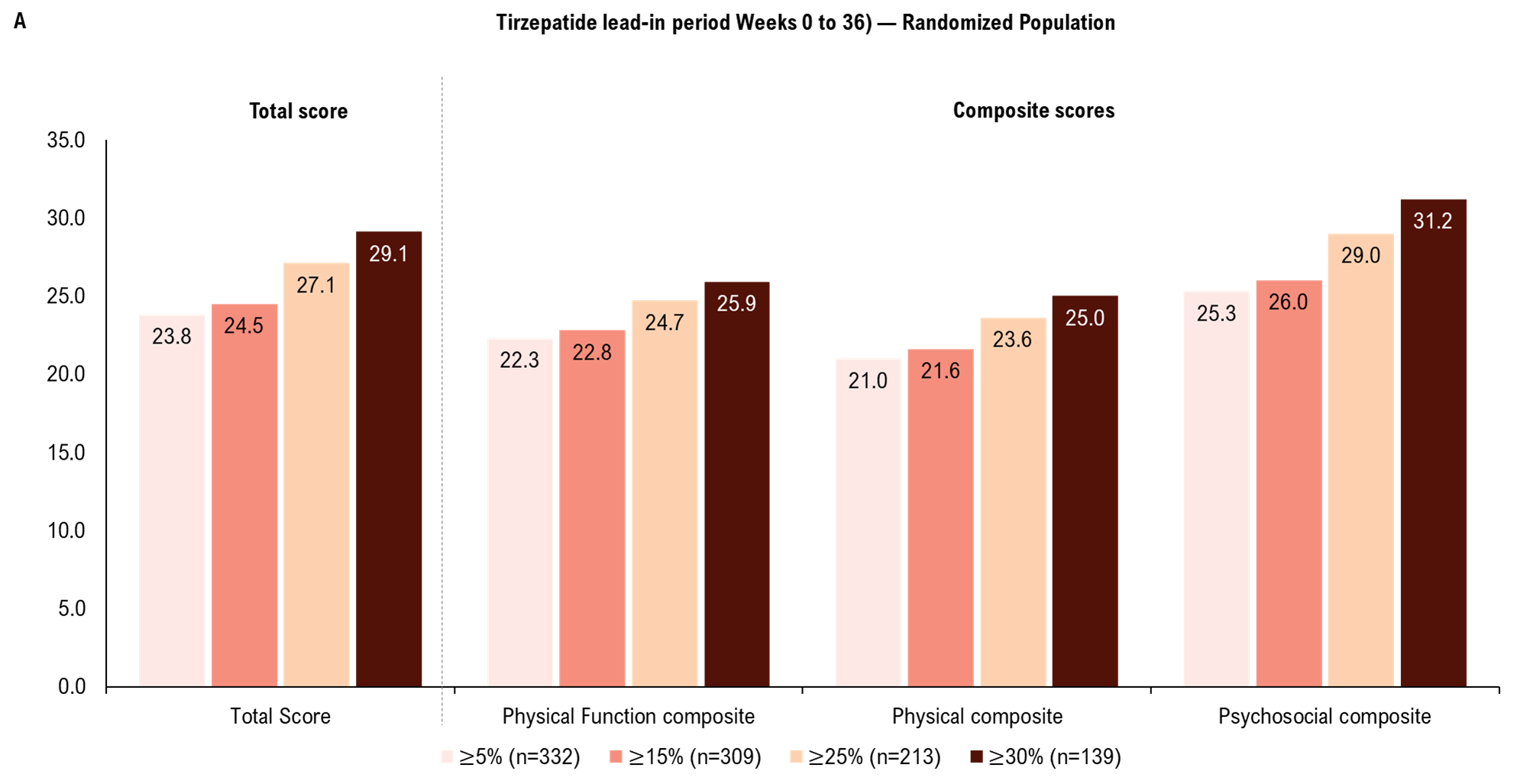


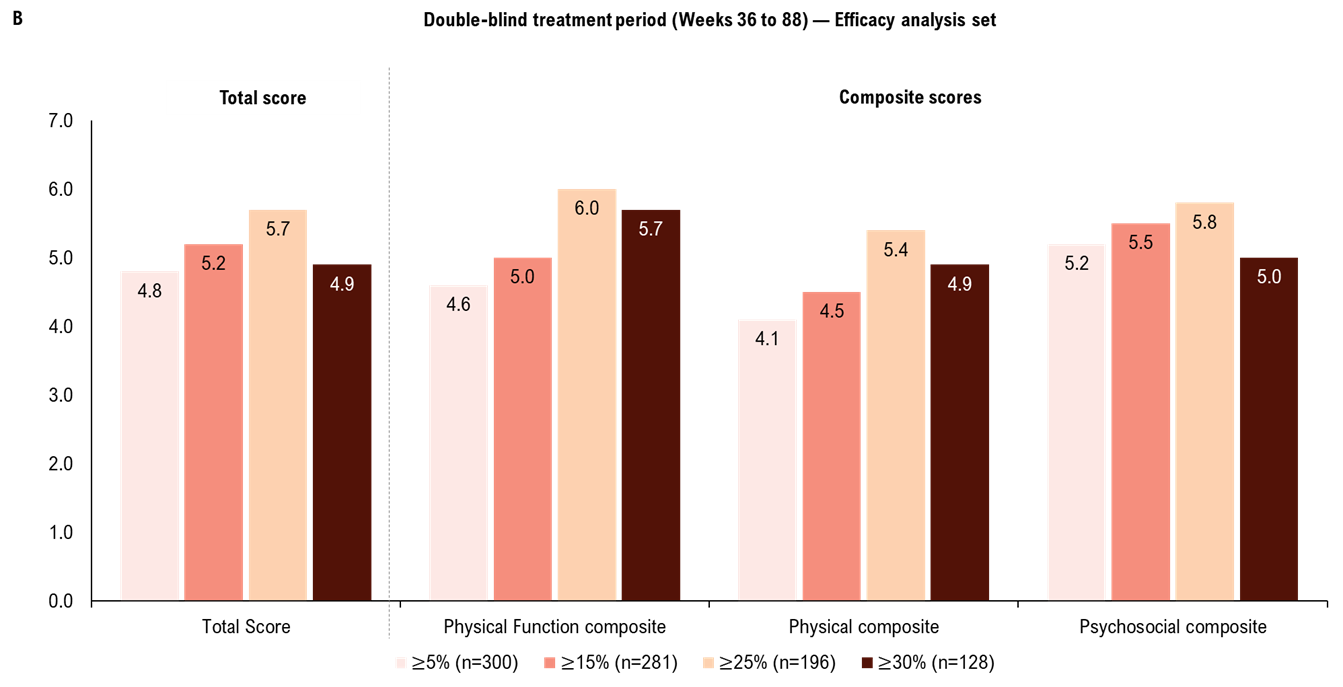


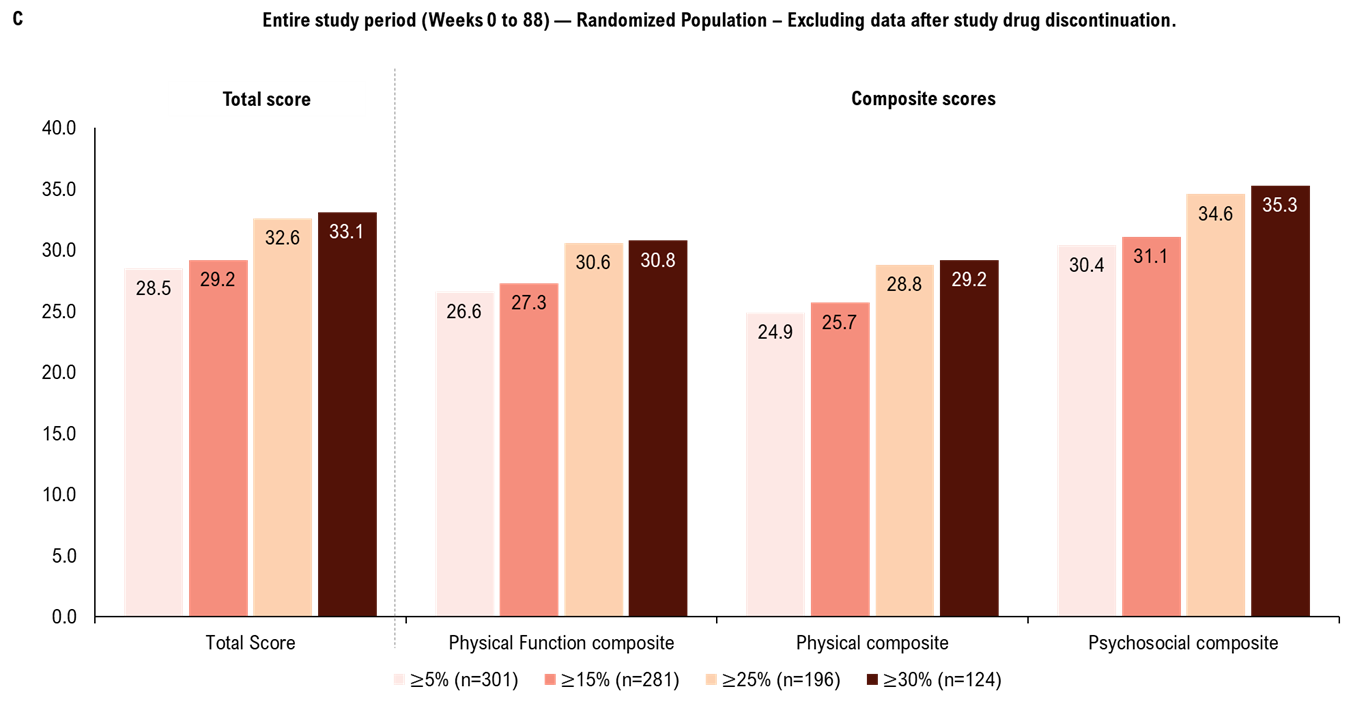


Data are presented as mean change from baseline (Week 0) or randomization (Week 36) to Week 88 in patient-reported outcomes using last observation carried forward.

Scores are transformed to a scale of 0 to 100, with higher scores reflecting better levels of functioning.

Abbreviations: n, number of participants with non-missing baseline and at least 1 non-missing post-baseline value
